# Assessment of Metabolic Alterations Induced by Halogenated Additives and Antifungal Activity of Extracts from the Endophytic Fungus *Fusarium* sp. Associated with *Dizygostemon riparius* (Plantaginaceae)

**DOI:** 10.3390/metabo15070451

**Published:** 2025-07-04

**Authors:** Hilzimar de Jesus Freitas Sá, Anne Karoline Maiorana Santos, Adriano Souza Fonseca, Lourivaldo da Silva Santos, Josivan Regis Farias, Rosane Nassar Meireles Guerra, Edson Rodrigues-Filho, Gilmar Silverio da Silva, Cleydlenne Costa Vasconcelos, Alberto Jorge Oliveira Lopes, Antônio José Cantanhede Filho

**Affiliations:** 1 Chemistry Postgraduate Program, Federal Institute of Science Education and Technology of Maranhão, São Luís 65030-005, Brazil; 2 Institute of Chemistry of Araraquara, São Paulo State University, Araraquara 14800-060, Brazil; 3 Institute of Exact and Natural Sciences, Federal University of Pará, Belém 66075-110, Brazil; 4 Center for Biological and Health Sciences, Federal University of Maranhão, São Luís 65030-005, Brazil; 5 Micromolecular Biochemistry of Microorganisms Laboratory (LaBioMMi), Department of Chemistry, Center for Exact Sciences and Technology, Federal University of São Carlos, São Carlos 13563-120, Brazil

**Keywords:** secondary metabolites, biofilm inhibition, natural product discovery, halogen and metal supplementation

## Abstract

**Background/Objectives**: Endophytic fungi are valuable sources of bioactive compounds with potential therapeutic applications. This study aimed to evaluate the antifungal activity of secondary metabolites produced by *Fusarium* sp. isolated from *Dizygostemon riparius*, with particular focus on the impact of culture medium supplementation with halogenated and metallic additives on metabolite production. **Methods**: The fungus was cultivated in standard Czapek medium and media supplemented with NH_4_Br or MnCl_2_. Methanolic extracts were obtained, fractionated, and chemically characterised via LC-ESI-HRMS. In vitro antifungal assays, including MIC and MFC determinations and biofilm inhibition tests, were performed against *Candida albicans* strains. In vivo toxicity and efficacy were assessed using *Tenebrio molitor* larvae. **Results**: Fifteen metabolites were annotated, including known antifungals such as fusaric acid and cyclosporin A. Fractions EMBr4 and EMC5 demonstrated fungicidal activity with MIC values close to fluconazole and significantly inhibited biofilm formation and maturation. In vivo, these fractions displayed low acute toxicity and improved survival in infected larvae, comparable to fluconazole treatment. **Conclusions**: The results indicate that culture medium modulation enhances the production of bioactive metabolites by *Fusarium* sp., leading to extracts with notable antifungal efficacy and safety. EMBr4 and EMC5 are promising candidates for further development as antifungal agents, particularly for targeting biofilm-associated *Candida* infections. These findings support the potential of endophytic fungi as sources of novel therapeutics and warrant further mechanistic and pharmacological investigations.

## 1. Introduction

The *Fusarium* genus displays remarkable diversity in morphological, physiological, and ecological traits, occupying a wide range of ecological niches across diverse geographical regions. Recent molecular phylogenetic analyses have identified over 400 genetically distinct species within the genus, organised into 23 monophyletic lineages referred to as species complexes. These fungi thrive particularly in tropical and subtropical climates and possess the capacity to persist in soil for extended periods through the formation of chlamydospores. In addition, *Fusarium* species can colonise roots, leaves, inflorescences, and fruits via their conidia, which are dispersed through air or water. This adaptability is further supported by the complexity of soil ecosystems, the diversity of species present, and the considerable genetic variability within the genus [1,2,3].

Fungi exhibit a remarkable ability to reproduce and adapt under diverse nutritional conditions by modulating their metabolism according to nutrient availability in the culture medium. This metabolic flexibility enables the induction of microbial biosynthesis of compounds of biotechnological interest [4]. Previous studies have demonstrated that alterations in cultivation parameters can significantly impact microbial metabolic profiles [5,6,7].

The OSMAC (One Strain Many Compounds) approach aims to explore these adaptive responses using tools such as bioinformatics and advanced analytical and biochemical methodologies. This strategy has proven to be a straightforward and promising means of enhancing the production of potentially bioactive secondary metabolites by modifying fundamental culture conditions, such as medium composition or the inclusion of epigenetic modifiers. Unlike traditional screening methods that rely on a single strain under fixed conditions, OSMAC leverages the metabolic versatility of microorganisms to maximise their biosynthetic potential [8].

Metals play a pivotal role in microbial physiology by acting as cofactors in various biochemical processes, including transcriptional regulation, morphological differentiation, and metabolic development. Moreover, they can activate specific biosynthetic pathways. Manganese (Mn), iron (Fe), and copper (Cu) are of particular interest due to their redox properties and catalytic activity, which are essential for several biological functions. These metals commonly exhibit a stable +2 oxidation state but may also shift between multiple states (up to seven in the case of Mn), enabling redox cycling during enzymatic reactions [9]. This redox flexibility underscores their importance in enzymatic catalysis and metabolic adaptability, offering potential applications in biotechnology, including the optimisation of microbial production systems and the engineering of biosynthetic pathways for pharmaceutical or industrial purposes.

Biotechnological studies have shown that *Fusarium* sp. secretes a wide array of enzymes, including xylanases, cellulases, pectinases, and lipases, in addition to various secondary metabolites such as pigments [10,11,12]. For instance, *Fusarium moniliforme* produces pigments like bikaverin and carotenoids, which have demonstrated bioactivity against *Leishmania braziliensis* [13]. Among human pathogens, *Candida albicans* is one of the most reported species and has been classified by the World Health Organization (WHO) as a critical-priority pathogen due to its multiple invasion mechanisms and association with severe infections [14,15,16,17]. Therefore, the aim of the present study was to investigate the antifungal potential of secondary metabolites produced by endophytic fungi isolated from *Dizygostemon riparius* against *Candida albicans* strains. In addition, particular attention was given to the role of metal ions in optimising the production of bioactive compounds in the culture medium, contributing to the development of novel antifungal therapies.

## 2. Materials and Methods

### 2.1. Endophytic Fungus

The original fungal strain of *Fusarium* sp. under code MA-O1 was obtained from the culture collection of the Federal Institute of Maranhão. It was isolated from the leaves of *Dizygostemon riparius* (Plantaginaceae) collected in the municipality of São Benedito do Rio Preto (3°20′02″ S 43°31′40″ W), Maranhão, BR, in 2019.

### 2.2. Fermentation and Extraction

The *Fusarium* sp. fungal strain was reactivated on a PDA plate at 25 °C for 7 days. The inoculum was prepared individually from 0.5 cm × 0.5 cm cuts in three different cultivation conditions [5]. In the first condition, the fungus was cultivated in 20 × 500 mL Erlenmeyer flasks containing 150 mL of liquid Czapek medium composed of NaNO_3_ (3.0 g L^−1^); K_2_HPO_4_ (1.0 g L^−1^); MgSO_4_ (0.5 g L^−1^); KCl (0.5 g L^−1^); FeSO_4_‧7H_2_O (0.01 g L^−1^); Glucose (30.0 g L^−1^); and yeast extract (20.0 g L^−1^). The second and third conditions were similar, but with the addition of NH_4_Br (15 mMol) and MnCl_2_ (10 mMol), respectively. The fermentation was maintained for 21 days under static conditions at room temperature [5].

At the end of the fermentation for each condition, the mycelium was subsequently extracted with MeOH (150 mL) and left to rest for 24 h, followed by filtration and evaporation of the solvent under reduced pressure, producing the crude ethanolic extracts of the Control (Czapek), NH_4_Br, and MnCl_2_, named respectively as CM, MBr, and MMn. After obtaining the extracts in MeOH, their chemical profiles were evaluated by TLC, HPLC, and UPLC-ESI-QTOF-MS.

### 2.3. Microculture and Morphological Identification

The isolated colonies were cultured using the streaking technique for subsequent identification, which was performed based on macro- and micromorphological characteristics. The macromorphological characteristics evaluated included colony size, margin features, texture, elevation, and pigmentation to distinguish the selected fungus from others present. Micromorphological analyses were conducted using the microculture technique on BDA medium. For this technique, a system was developed resembling a “sandwich” setup within a Petri dish: a lamella, a cube of BDA medium, and a cover slip were placed over a V-shaped straw section as a support, with sterile distilled water-soaked cotton providing humidity. Adaptations were made to enhance microscopic visualization, which was performed after 10 days of microculture growth. The upper cover slips were stained with cotton blue dye and immediately observed under a microscope. If the structural characteristics of the fungus were not visible on the upper cover slip, the lower cover slip was examined to identify vegetative and, specifically, reproductive structure characteristics of the fungal genus [18].

### 2.4. Extraction of Crude Extracts and Fractionation of Methanolic Extracts from Control (Czapek) and Media Modified with MnCl_2_ and NH_4_Br

After 21 days of fungal fermentation in three types of culture media—standard Czapek (Control), Czapek supplemented with MnCl_2_, and Czapek supplemented with NH_4_Br—the mycelial mass was separated from the culture medium by vacuum filtration. The methanolic extract (E.MeOH) was prepared by macerating the fungal mycelium in 150 mL of methanol. The mixture was then filtered, and the extract was concentrated using a rotary evaporator [19]. Fractionation of the methanolic extracts (E.MeOH from the standard Czapek and modified media (MnCl_2_ and NH_4_Br)) was performed using open-column chromatography. Silica gel 60 (Merck, New York, USA, 0.015–0.040 nm) was used as the stationary phase. The mobile phase consisted of hexane, ethyl acetate, and methanol, along with binary mixtures of hexane/ethyl acetate and ethyl acetate/methanol, applied in an order of increasing polarity. Eight of each fraction were obtained, and the elution systems are detailed in Table S1, Supplementary Material.

### 2.5. Chromatographic Profile by High-Performance Liquid Chromatography (HPLC) of the Obtained Fractions

The obtained fractions were analysed by comparative thin-layer chromatography (TLC), using hexane, ethyl acetate, methanol, and mixtures thereof as eluents, in order of increasing polarity, to evaluate possible metabolic differentiations under different culture media. High-Performance Liquid Chromatography (HPLC) analysis was performed using a Diode Array Detector (DAD) with the following conditions and parameters: flow rate of 0.9 mL/min, temperature of 28 °C, and a C18 column (Luna model, Phenomenex brand, dimensions: 250 mm × 4.6 mm × 5 μm). A gradient elution mode was employed, with Mobile Phase A consisting of 0.01% formic acid in water and Mobile Phase B consisting of 0.01% formic acid in methanol. The gradient program was as follows: 0 min: 5% B; 70 min: 100% B; 100 min: 100% B; 105 min: 5% B. These conditions indicate the use of a gradient elution, where the methanol concentration (Phase B) is gradually increased over time to elute the sample compounds from the C18 column. The specific gradient programming, with an increase from 5% to 100% B over 70 min, maintenance at 100% B for 30 min, and re-equilibration to 5% B, ensures efficient separation of the compounds [5].

### 2.6. Analysis by Ultra-Performance Liquid Chromatography—Electrospray Ionisation—Quadrupole Time-of-Flight Mass Spectrometry (UPLC-ESI-QTOF-MS)

The fractions EMC5, EMBr4, and EMMn3, derived from methanolic extracts of both the modified and control groups, were selected for this analysis due to their promising preliminary bioactivity and potential to yield significant results. The solvents used in the chromatographic processes were UPLC-grade LiChrosolv^®^. The water was distilled and deionized using a Milli-Q system (minimum resistivity of 18.2 MΩ·cm at 25 °C). The samples were prepared for UPLC-QTOF-MS analysis by dissolving them in a hydro-methanolic solution [MeOH:H_2_O (95:5)] at 10 mg/mL and subjecting them to a solid-phase extraction (clean-up) process using a Sep-Pak cartridge (SPE, Macherey-Nagel C18ec, 3.0 mL, 500 mg, Chromabond^®^). The samples were evaporated, reconstituted in UPLC-grade acetonitrile, and prepared at a concentration of 500 ppm. UPLC-ESI-QTOF-MS analyses were performed using the Xevo G2-S Q-TOF system (Waters Corporation) under the following parameters: mobile phase A was water with 0.1% formic acid (*v*/*v*), and mobile phase B was acetonitrile with 0.1% formic acid (*v*/*v*). The method employed a linear gradient as follows: 5–100% B for 8 min, 100% B maintained from 8–14 min, and a return to the initial condition of 5% B from 14–20 min. The flow rate was 0.45 mL/min, the injection volume was 0.2 μL, and the stationary phase was a reverse-phase C18 Acquity UPLC^®^ HSS T3 Column (1.8 μm; 2.1 mm × 100 mm) maintained at 35 °C [5].

Spectra were acquired in positive mode over an *m*/*z* range of 100–1500 operating in Auto (MS) mode. Data-dependent acquisition (DDA) analyses were conducted with the following parameters: capillary voltage of 2.5 kV; low mass collision energy ramp: start at 10 eV and end at 30 eV; high mass collision energy ramp: start at 50 eV and end at 70 eV; collision energy at 4 eV. The system switched to MS/MS mode when the intensity exceeded the threshold (500,000). Detected precursors were included with real-time exclusion and re-included after 5 s. Lock mass calibration was set to *m*/*z* 556.277100.

Data were converted from WATERS.raw format to mzML format using ProteoWizard V3 MSConvert software and processed using MZMine 2.53. To ensure optimal mass spectrometry conditions for all monitored analytes, each sample underwent two chromatographic runs under electrospray ionization (ESI) in both positive and negative modes, following the protocol of [20].

### 2.7. In Vitro and In Vivo Tests for Anti-Candida Activity

#### 2.7.1. Candida Strains, Growth Conditions, and Inoculum Preparation

Standard strains of *C. albicans* (ATCC 90028), along with clinical isolates, were sourced from the collection of the Laboratory of Immunophysiology at the Federal University of Maranhão, São Luís, MA, Brazil. The yeasts were reactivated on Sabouraud dextrose agar (Kasvi, Italy) for 24 h at 37 °C. The inoculum was prepared in a NaCl (0.85%) solution from colonies cultured for 24 h and adjusted using spectrophotometry (Global Trade Technology) at a wavelength of 530 nm to a cell density equivalent to McFarland standard 0.5. For the microdilution tests, the suspension was diluted in RPMI 1640 with glutamine, without bi-carbonate (Sigma-Aldrich, St. Louis, MO, USA), pH 7.0, and buffered with 3-(N-morpholino) propane-sulfonic acid (MOPS, Sigma Chemical, St. Louis, MO, USA) at a concentration of 1 × 10^3^ to 5 × 10^3^ CFU/mL ((CLSI), [21]).

#### 2.7.2. Determination of Minimum Inhibitory Concentration (MIC)

The susceptibility profile of the *C. albicans* strains tested against the fractions were standardized following criteria established by the Clinical and Laboratory Standards Institute (M27-A3) [21], with adaptations. For the assay, 100 µL of RPMI 1640 medium (with glutamine and phenol red, without bicarbonate) buffered with 3-(N-morpholino) propane-sulfonic acid (MOPS) was added to the wells of a sterile 96-well microtiter plate, which contained the standardized *Candida* inoculum suspensions. Subsequently, 100 µL of the chemical isolates at an initial concentration of 1000 µg/mL were added to the first column, followed by serial dilutions down to 1.953 µg/mL. Fluconazole (0.125–64 µg/mL) (Sigma-Aldrich, São Paulo, Brazil) was used as a positive control. RPMI 1640, without the test substances, was used as a negative growth control, and 2% (*v*/*v*) DMSO 2% was used as a vehicle control. The assay microplates were incubated for 48 h at 37 °C, and the outcome was visually analysed and read on a microplate reader at 540 nm. The MIC was defined as the lowest concentration of the extract or antifungal agent at which no growth was visible or detected. The test was performed in triplicate in two independent experiments.

#### 2.7.3. Determination of Minimum Fungicidal Concentration (MFC)

The MFC was determined based on the results found in the MIC assay. A 10 μL aliquot from the wells corresponding to up to 4× the MIC value was transferred to Petri dishes containing Sabouraud dextrose agar with chloramphenicol (Kasvi, Italy) [16]. The plates were incubated for 24 to 48 h at 37 °C, and the MFC was defined as the lowest concentration of the test substances that inhibited fungal growth in colonies. All tests were performed in triplicate in two independent experiments. The MFC/MIC ratio was calculated to determine whether the extract had fungistatic (MFC/MIC ≥ 4) or fungicidal (MFC/MIC ≤ 4) activity [22].

#### 2.7.4. Effect of Extract Against Candida Biofilms

The assays were conducted according to the inoculum standardization protocol described previously. Following this, the inoculum was cultivated in yeast nitrogen base (YNB; Sigma-Aldrich) supplemented with 50 mM glucose and incubated at 37 °C for 18 h. The yeast suspensions were centrifuged at 1666× *g* (Centrifuge 5810R, Eppendorf) for 5 min, and the cell pellet was washed twice with sterile PBS and then resuspended in 5 mL of sterile PBS. The optical densities of the inoculum were adjusted to 1 × 10^7^ CFU/mL. Moreover, 200 µL of each yeast suspension was transferred to 96-well microtitulation plates (Kasvi, Ortygia) and incubated for 90 min at 37 °C for the adhesion process. Then, the supernatants were aspirated, and the wells were washed twice with sterile PBS to remove non-adherent yeasts [23,24].

To evaluate the inhibitory effects of the compounds under study on young (pre-formed) biofilms, 200 µL of each compound at sub-inhibitory concentrations of 1/4 MIC and 1/2 MIC, diluted in YNB supplemented with 50 mM glucose, was added to each well with adherent cells, followed by incubation for 24 h at 37 °C. After incubation, the super-natant was aspirated, the former biofilms were washed twice with sterile PBS, and then their viability was determined by the 3-(4,5-dimethylthiazol-2-yl)-2,5-diphenyltetrazolium bromide (MTT) assay (Sigma-Aldrich, St. Louis, MO, USA) and their biomass was defined by crystal violet staining; both methodologies are described in subsequent sections. In the analysis of inhibitory effects on formed biofilms, 200 µL of each compound at concentrations above the MIC (2× MIC and 4× MIC) diluted in YNB supplemented with 50 mM glucose was added to each well with adherent cells, followed by incubation for 48 h at 37 °C.

After incubation, the supernatant was aspirated, the former biofilms were washed twice with sterile PBS, and then their viability was determined by the MTT assay and their biomass defined by crystal violet staining. For the analysis of inhibitory effects on mature (formed) biofilms, the inoculum was incubated in microplates containing YNB supplemented with 50 mM glucose for 48 h, with the medium being changed at the 24 h mark. Afterwards, the supernatants were aspirated, the biofilms were washed twice with sterile PBS, and 200 µL of the test substances at concentrations above the MIC (2× MIC and 4× MIC) was added. The microplates were incubated for an additional 24 h at 37 °C. After the incubation period, the supernatants were aspirated, and the biofilms were washed twice with sterile PBS and evaluated by MTT and crystal violet staining. In all experiments, biofilms without test substances and biofilms with 2% DMSO were used as negative controls, and fluconazole was used as a positive control. For both assays, triplicates were performed in two independent experiments.

#### 2.7.5. Biofilm Viability Assay

The metabolic activity of fungal cells within the biofilm was determined using the MTT method [25] with some modifications [15,16]. After the biofilm washing process, 100 µL of MTT solution (5 mg/mL; Sigma, USA) in YNB medium supplemented with 50 mM glucose was added to each sample, which was then incubated at 37 °C for 4 h in the dark. Subsequently, the supernatants were removed. Then, 100 µL of DMSO was added to each well, and the samples were incubated for an additional 10 min. The absorbance of the plate was read on a microplate reader (Softmax^®^ Pro; San Jose, CA, USA) at 570 nm.

#### 2.7.6. Crystal Violet Staining Analysis of Biofilm

The biofilms were quantified using the crystal violet staining method [26]. After the biofilms were washed with sterile PBS, they were air-dried at room temperature and fixed and resuspended in 200 µL of 95% (*v*/*v*) methanol, then incubated for 15 min at 37 °C. The methanol was completely removed, and the plates were air-dried for 20 min at room temperature. Subsequently, 200 µL of crystal violet solution (1% *v*/*v*) was added to each well, and the samples were incubated with the dye for 5 min. The plates were washed twice with sterile PBS, and 200 µL of acetic acid (33% *v*/*v*) was added to each well. To obtain absorbance values, 100 µL from each of the sample wells was transferred to a new 96-well microplate and read at 570 nm on the microplate reader.

### 2.8. In Vivo Assay in Tenebrio molitor Larvae

Larvae of *T. molitor* (Coleoptera, Polyphaga: Tenebrionidae) at early stages, 11th to 12th, and weighing approximately 200 mg, were obtained from specialized breeders and then selected based on size similarity and exhibiting no apparent colour alterations for use in all experiments. The larvae were placed in sterile Petri dishes for 24 h prior to the experiments for acclimatization and incubated at 37 °C, protected from light. Larvae dis-playing dark spots or apparent melanisation processes were excluded [5,27].

#### 2.8.1. Acute Toxicity Assay Against *T. molitor* Larvae

Extracts at concentrations of 2×, 4×, and 8× MIC (µg/kg) were injected in 5 µL using a Hamilton syringe (Hamilton, FL, USA) via the intrahemocoelic route, previously cleaned with 70% alcohol, between the fourth and fifth sternites of the lower ventral abdomen of the larvae (n = 30 larvae/group). The control groups consisted of larvae that received sterile PBS and 2% DMSO. Subsequently, the larvae were incubated at 37 °C, and deaths were assessed every 24 h for 10 days. Death was defined as the complete loss of movement and absence of response to physical stimuli and an intense melanisation process [5]. The assays were repeated three times in two independent experiments.

#### 2.8.2. Efficacy of Extracts in *T. molitor* Larvae Infected with *C. albicans*

Strains of *C. albicans* were cultivated on Sabouraud dextrose agar (Kasvi, Italy) for 24 h at 37 °C. The inoculum was standardized according to Silva et al. [15], with a concentration of 5 × 10^5^/5 µL considered lethal. For this assay, treatment concentrations corresponding to 2× MIC and 4× MIC (µg/kg) were used, relating to the inhibition of the standard strains by the chemical isolates and fluconazole. The larvae (n = 30 larvae/group) received 5 µL of the standard inoculum via the previously described route and region, then were placed in Petri dishes and incubated at 37 °C. After a period of 3 h, the larvae received a single dose of 5 µL of the test concentrations, and the plates were again incubated at 37 °C. The control groups corresponded to sham (larvae without intervention), control (infected larvae treated with sterile PBS), and 2% DMSO (infected larvae treated with 2% DMSO). Mortality rates for each group were determined every 24 h for 10 days, with deaths evaluated by the criteria previously mentioned. The assays were repeated three times in two independent experiments.

### 2.9. Statistical Analysis

The GraphPad Prism 9.0 software (La Jolla, CA, USA) was used for graphical and statistical purposes. Results are expressed as mean ± standard deviation. Statistical analysis was performed using the Student’s *t*-test. Survival curves were analysed using the log-rank test. A *p*-value < 0.05 was considered statistically significant.

## 3. Results

### 3.1. Annotation of Compounds Present in the MeOH Extracts from Mycelium

Based on analyses performed using UPLC-ESI-QTOF-MS in both positive and negative ionisation modes, 16 compounds were annotated from the methanolic extracts—EMC5, EMBr4, and EMMn3—as summarised in Table 1 and illustrated below. The total ion chromatogram (TIC) for each fraction is presented in the Supplementary Material (Figures S1–S5). Additionally, the extracted ion chromatograms (EICs) corresponding to each annotated compound are provided in the Supplementary Material (Figures S6–S21).

**Figure 1:**
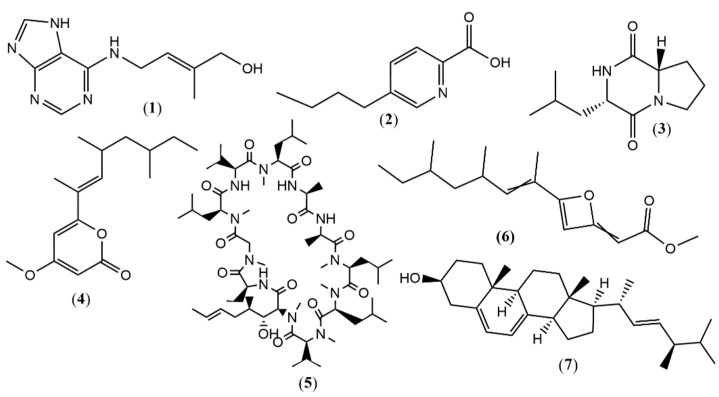
Compounds annotated by UPLC-ESI-QTOF-MS in positive and negative modes for EMC5 fraction.

In the experimental control (EMC5), obtained from fractionation in column chromatography with 1:1 (EtOAc/MeOH) eluent, seven compounds (Figure 1) were annotated as zeatin (**1**), *m*/*z* 220.1182 ([M+H]^+^, calc. 220.1193 for C_10_H_14_N_5_O^+^, ∆ = −4.99 ppm); fusaric acid (**2**), *m*/*z* 180.1026 ([M+H]^+^, calc. 180.1019 for C_10_H_14_NO_2_^+^, ∆ = 3.88 ppm); L-Leucyl-L-proline lactam (**3**), *m*/*z* 211.1441 ([M+H]^+^, calc. 211.1441 for C_11_H_19_N_2_O_2_^+^, ∆ = 0.00 ppm); 4-methoxy-6-((*E*)-4,6-dimethyloct-2-en-2-yl)-2H-pyran-2-one (**4**), *m*/*z* 265.1805 ([M+H]^+^, calc. 265.1798 for C_16_H_25_O_3_^+^, ∆ = 2.64 ppm); cyclosporin A (**5**) *m*/*z* 1202.8492 ([M+H]^+^, calc. 1202.8486 for C_62_H_112_N_11_O_12_^+^, ∆ = 0.49 ppm); fusariumin D (**6**) *m*/*z* 265.1805 ([M+H]^+^, calc. 265.1798 for C_16_H_25_O_3_^+^, ∆ = 2.63 ppm); and ergosterol (**7**) *m*/*z* 397.3470 ([M+H]^+^, calc. 397.3465 for C_28_H_45_O^+^, ∆ = 1.25 ppm).

**Figure 2:**
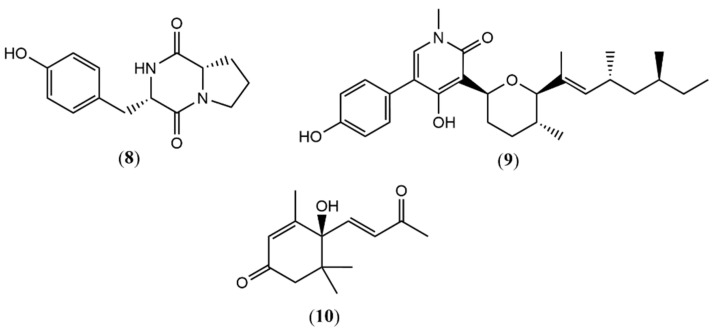
Compounds annotated by UPLC-ESI-QTOF-MS in positive and negative modes for EMBr4 fraction.

Compounds 8–10 (Figure 2) were founded in the MeOH extracts from the NH_4_Br -supplemented media (EMBr4), obtained from fractionation in column chromatography with 7:3 (EtOAc/MeOH) eluent and annotated as cyclo(Pro-Tyr) (**8**) *m*/*z* 261.1234 ([M+H]^+^, calc. 261.1234 for C_14_H_17_N_2_O_3_^+^, ∆ = 0.00 ppm); (-)-sambutoxin (**9**) *m*/*z* 454.2937 ([M+H]^+^, calc. 454.2952 for C_28_H_40_NO_4_^+^, ∆ = −3.30 ppm); and Dehydrovomifoliol (**10**) *m*/*z* 221.1173 ([M-H]^-^, calc. 221.1183 for C_13_H_17_O_3_^-^, ∆ = −4.52 ppm).

**Figure 3:**
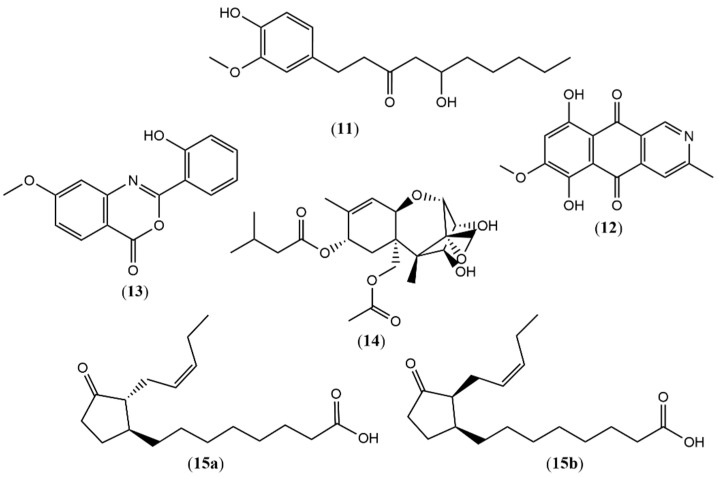
Compounds annotated by UPLC-ESI-QTOF-MS in positive and negative modes for EMMn3 fraction.

Additionally, from MnCl_2_ media (EMMn3) obtained from fractionation in column chromatography with 9:1 (EtOAc/MeOH) eluent, six more compounds (Figure 3) were annotated as 5-hydroxy-1-(4-hydroxy-3-methoxyphenyl)decan-3-one (**11**) *m*/*z* 293.1781 ([M-H]^-^, calc. 293.1758 for C_17_H_25_O_4_^-^, ∆ = 7.84 ppm); bostrycoidin (**12**) *m*/*z* 286.0704 ([M+H]^+^, calc. 286.0710 for C_15_H_12_NO_5_^+^, ∆ = −2.09 ppm); methoxydianthalexin S (**13**) *m*/*z* 270.0767 ([M+H]^+^, calc. 270.0761 for C_15_H_12_NO_4_^+^, ∆ = 2.22 ppm); HT-2 toxin (**14**) *m*/*z* 425.2144 ([M+H]^+^, calc. 425.2170 for C_22_H_33_O_8_^+^, ∆ = −6.11 ppm); (1S,2R)-3-Oxo-2-(2Z-pentenyl) cyclopentane-1-octanoic acid (**15a**) *m*/*z* 295.2267 ([M+H]^+^, calc. 295.2268 for C_18_H_31_O_3_^+^, ∆ = −0.33 ppm); and (1S,2S)-3-Oxo-2-(2Z-pentenyl) cyclopentane-1-octanoic acid (**15b**) ([M+H]^+^, calc. 295.2268 for C_18_H_31_O_3_^+^, ∆ = −0.33 ppm).

### 3.2. In Vitro Anti-Candida Assays

#### 3.2.1. Antifungal Activity of *Fusarium* sp. Extract Fractions Against Candida Albicans Strains

Table 2 summarises the minimum inhibitory concentrations (MICs) and minimum fungicidal concentrations (MFCs) of three fractions obtained from the endophytic fungus *Fusarium* sp., tested against five *Candida albicans* strains, comprising two reference strains (ATCC 90028 and ATCC 10231) and three clinical isolates (A1–A3).

For the ATCC 90028 strain, the EMBR4 fraction exhibited the strongest inhibitory effect, with an MIC of 4.19 µg/mL and an MFC of 15.62 µg/mL (MFC/MIC ratio = 3.17). The EMMN3 fraction showed a slightly higher MIC (11.04 µg/mL), while EMC5 displayed an MIC of 15.61 µg/mL. All fractions presented MFC/MIC ratios ranging between 3.17 and 4.00, indicative of fungicidal potential.

Against the ATCC 10231 strain, EMMN3 and EMBR4 exhibited higher MICs (15.62 and 99.21 µg/mL, respectively), whereas EMC5 showed the highest MIC and MFC values (111.36 and 445.44 µg/mL), suggesting reduced efficacy against this strain.

Among the clinical isolates, EMC5 demonstrated notable activity against strain A1 (MIC = 8.76 µg/mL; MFC/MIC = 2.83), while EMBR4 exhibited enhanced efficacy against A2 (MIC = 31.24 µg/mL). In contrast, EMMN3 displayed weak activity against strain A3 (MIC = 280.61 µg/mL), with an unusually low MFC/MIC ratio (0.49), warranting further investigation into its mode of action.

Fluconazole, employed as the reference drug, consistently exhibited the lowest MIC values across all tested strains, ranging from 0.35 to 1.41 µg/mL, thereby validating the reliability of the experimental assays.

#### 3.2.2. Inhibition of Biofilm Viability by *Fusarium* sp. Extract Fractions in *Candida albicans*

**Figure 4:**
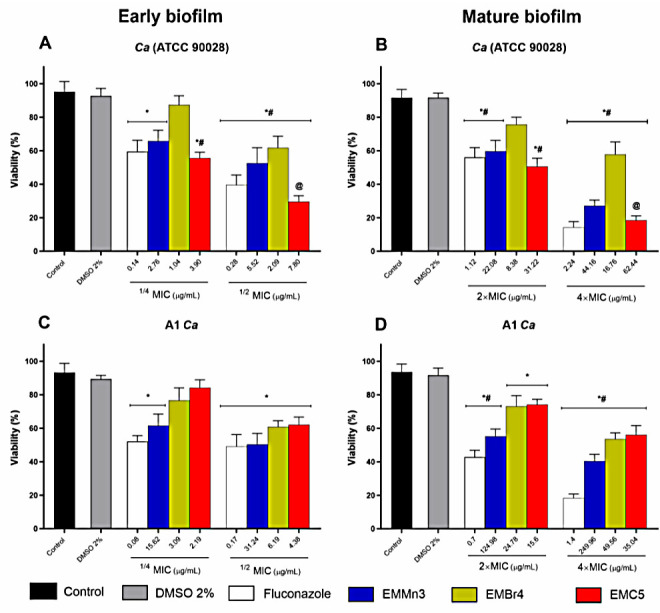
Percentage inhibition of metabolic activity in early-stage (**A**,**C**) and mature (**B**,**D**) *Candida albicans* biofilms following treatment with the EMMn3, EMBr4, and EMC5 fractions derived from the endophytic fungus *Fusarium* sp. Panels A and B correspond to the reference strain ATCC 90028, while panels C and D refer to the clinical isolate A1. Developing biofilms were treated with sub-inhibitory concentrations (1/4× and 1/2× MIC) and mature biofilms with supra-inhibitory concentrations (2× and 4× MIC). The MTT assay was employed to assess metabolic activity. Results were compared with both untreated controls and the reference antifungal fluconazole. (*) *p* < 0.05 vs. untreated control; (#) *p* < 0.005 vs. untreated control; (@) *p* < 0.05 vs. fluconazole.

Figure 4 illustrates the percentage inhibition of biofilm viability in both standard and clinical *Candida albicans* strains following treatment with the *Fusarium* sp. extract fractions EMMn3, EMBr4, and EMC5. Sub-inhibitory concentrations (1/4× and 1/2× MIC) significantly reduced the metabolic activity of developing biofilms in the ATCC 90028 strain (Figure 4A) and in the clinical isolate A1 (Figure 4C), as determined by the MTT assay. Notably, the EMBr4 and EMC5 fractions demonstrated greater inhibitory effects at 1/2× MIC than the reference antifungal fluconazole in both strains. Regarding mature biofilms (Figure 4B,D), all fractions effectively reduced viability at 2× and 4× MIC. Fraction EMC5 was particularly noteworthy, producing the greatest reduction in metabolic activity in mature biofilms of the clinical strain at 4× MIC, exceeding the effect observed with fluconazole. Statistical significance was observed in comparisons with both untreated controls and fluconazole-treated groups (*p* < 0.05; # *p* < 0.005; @ *p* < 0.05).

**Figure 5:**
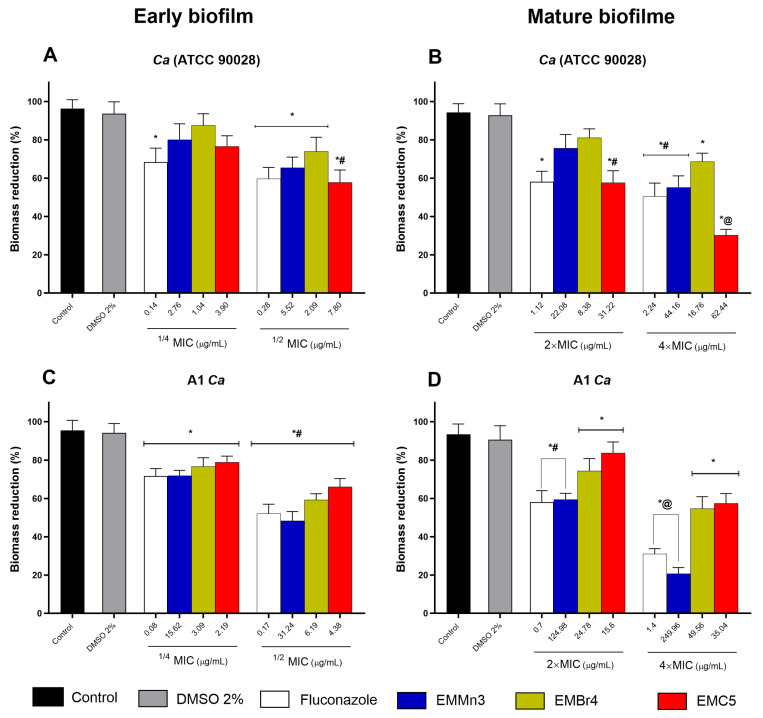
Percentage reduction in biomass of young (**A**,**C**) and mature (**B**,**D**) *Candida albicans* biofilms treated with fractions EMMn3, EMBr4, and EMC5 derived from *Fusarium* sp. Panels A and B refer to the standard strain (ATCC 90028) and C and D to the clinical isolate A1. Treatments were based on 1/4× and 1/2× MIC for biofilm formation and 2× and 4× MIC for mature biofilms. Biomass quantification was performed using the crystal violet assay. Fluconazole was used as the reference antifungal. (*) *p* < 0.05 compared to untreated control; (#) *p* < 0.005 compared to untreated control; (@) *p* < 0.05 compared to fluconazole.

Figure 5 illustrates the reduction in biofilm biomass following treatment with the *Fusarium* sp. extract fractions EMMn3, EMBr4, and EMC5 in both the reference (*Candida albicans* ATCC 90028) and clinical (A1) strains. In early-stage biofilms (Figure 5A,C), treatment with sub-inhibitory concentrations (1/4× and 1/2× MIC) resulted in a marked decrease in biomass, particularly with the EMBr4 and EMC5 fractions at 1/2× MIC, demonstrating comparable or superior activity to fluconazole.

In mature biofilms (Figure 5B,D), EMBr4 and EMC5 were again the most effective, achieving substantial biomass reduction at 4× MIC. Notably, EMC5 displayed greater efficacy than the reference antifungal in the clinical isolate.

Statistical analyses revealed significant differences in biomass reduction relative to untreated controls and fluconazole-treated groups (*p* < 0.05; # *p* < 0.005; @ *p* < 0.05).

### 3.3. In Vivo Assays

#### 3.3.1. In Vivo Toxicity of *Fusarium* sp. Extract Fractions in *Tenebrio molitor* Larvae

**Figure 6:**
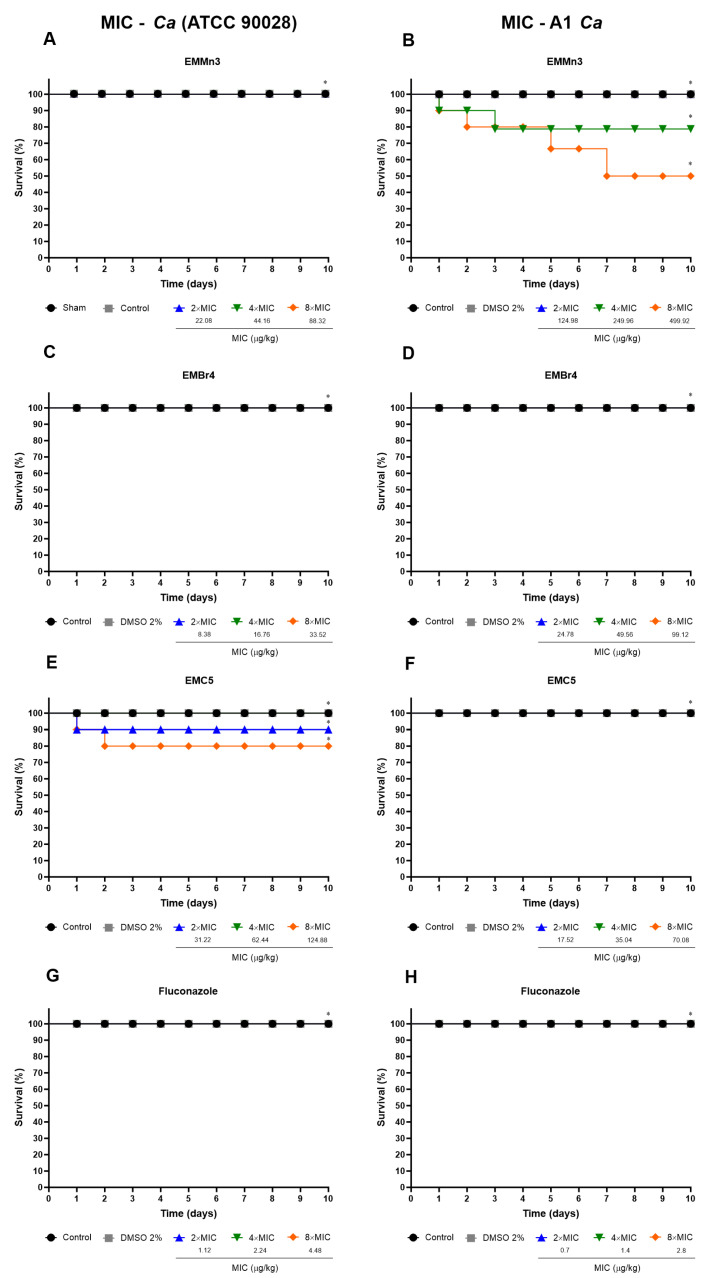
Evaluation of acute toxicity and survival percentage following injection of test fractions EMMn3 (**A**,**B**), EMBr4 (**C**,**D**), and EMC5 (**E**,**F**) derived from *Fusarium* sp. at concentrations of 2×, 4×, and 8× MIC (µg/kg) established for *Candida albicans.* Treatments were administered via intrahaemocoelic injection in *T. molitor* larvae. Fluconazole was used as the reference antifungal (**G**,**H**). Negative controls included larvae treated with sterile PBS and 2% DMSO. Survival was monitored over a 10-day period and assessed using the log-rank test. (*) *p* < 0.05 compared to the control group.

Figure 6 illustrates the survival rates of *Tenebrio molitor* larvae following treatment with extract fractions EMMn3, EMBr4, and EMC5 from *Fusarium* sp., administered at concentrations equivalent to 2×, 4×, and 8× the MIC values established for *Candida albicans*. At 2× MIC, all fractions exhibited low acute toxicity, with larval survival exceeding 80% throughout the 10-day observation period. Similarly, no toxicity was observed at 4× MIC, particularly in groups treated with EMBr4 and EMC5, where survival rates remained consistently above 85%. At 8× MIC, increased mortality was observed exclusively in the group treated with EMMn3, where survival dropped below 60%, with most deaths occurring within the first 4 days post-injection.

In contrast, EMBr4 and EMC5 maintained high survival even at this elevated concentration, indicating a favourable safety profile. The reference compound fluconazole, used as a positive control, demonstrated no significant toxicity, with survival consistently above 95% across all tested concentrations. Negative controls injected with PBS or 2% DMSO also exhibited high viability, further confirming the robustness of the experimental conditions.

#### 3.3.2. Survival of *T. molitor* Larvae Infected with *C. albicans* and Treated with *Fusarium* sp. Fractions

**Figure 7:**
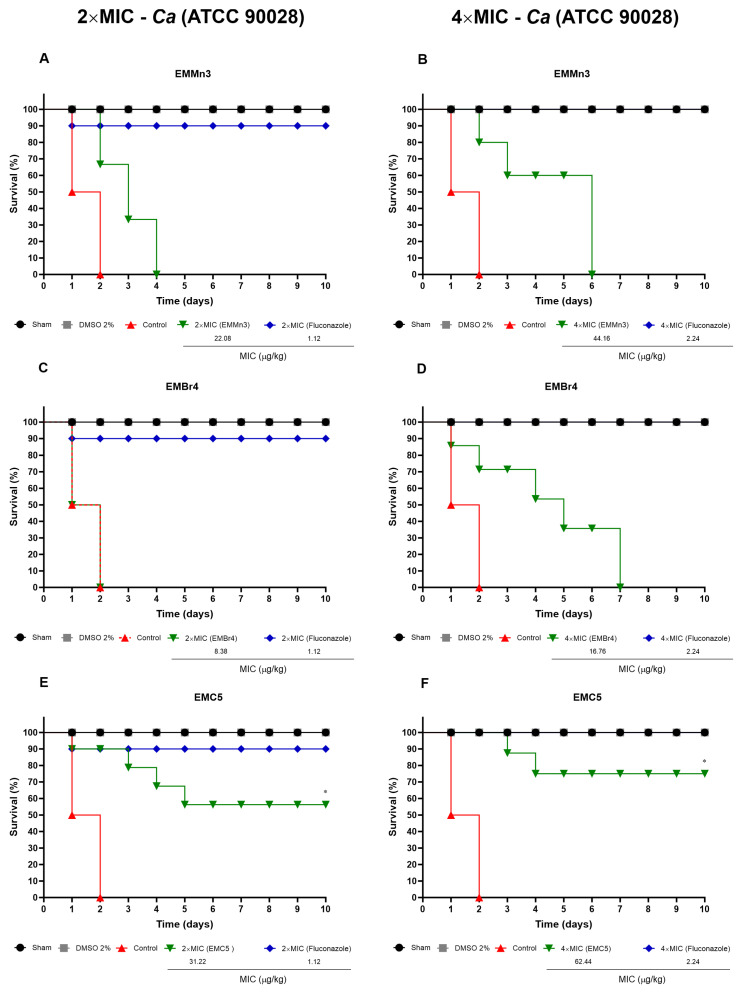
Survival percentage of *T. molitor* larvae after infection with *Candida albicans* (ATCC 90028) and treatment with *Fusarium* sp. extract fractions EMMn3 (**A**,**B**), EMBr4 (**C**,**D**), and EMC5 (**E**,**F**) at concentrations of 2× MIC (**A**,**C**,**E**) and 4× MIC (**B**,**D**,**F**) (µg/kg). Negative controls consisted of untreated infected larvae or those treated with sterile PBS and 2% DMSO. Fluconazole was used as the reference antifungal. Survival was monitored over a 10-day period and analysed using the log-rank test. (*) *p* < 0.05 compared to the control group.

Figure 7 presents the survival rates of *Tenebrio molitor* larvae following infection with *Candida albicans* (ATCC 90028) and subsequent treatment with the *Fusarium* sp. extract fractions EMMn3, EMBr4, and EMC5, administered at 2× and 4× MIC concentrations (µg/kg). In the groups treated with the EMMn3 fraction (Figure 7A,B), survival was notably reduced, particularly at 4× MIC, where cumulative mortality reached approximately 60% by day 6.

In contrast, treatment with EMBr4 (Figure 7C,D) led to improved survival, reaching 90% at 2× MIC and 80% at 4× MIC. The EMC5 fraction (Figure 7E,F) provided the greatest protection, with survival rates of 95% and 90% at 2× and 4× MIC, respectively—closely mirroring the efficacy of fluconazole. These results suggest a concentration-dependent protective effect, especially for EMBr4 and EMC5.

Larvae in the fluconazole-treated groups maintained survival rates above 90%, comparable to the uninfected controls. Conversely, negative controls (infected but untreated or treated with 2% DMSO) exhibited significantly higher mortality, thereby confirming the therapeutic efficacy of the tested fractions.

**Figure 8:**
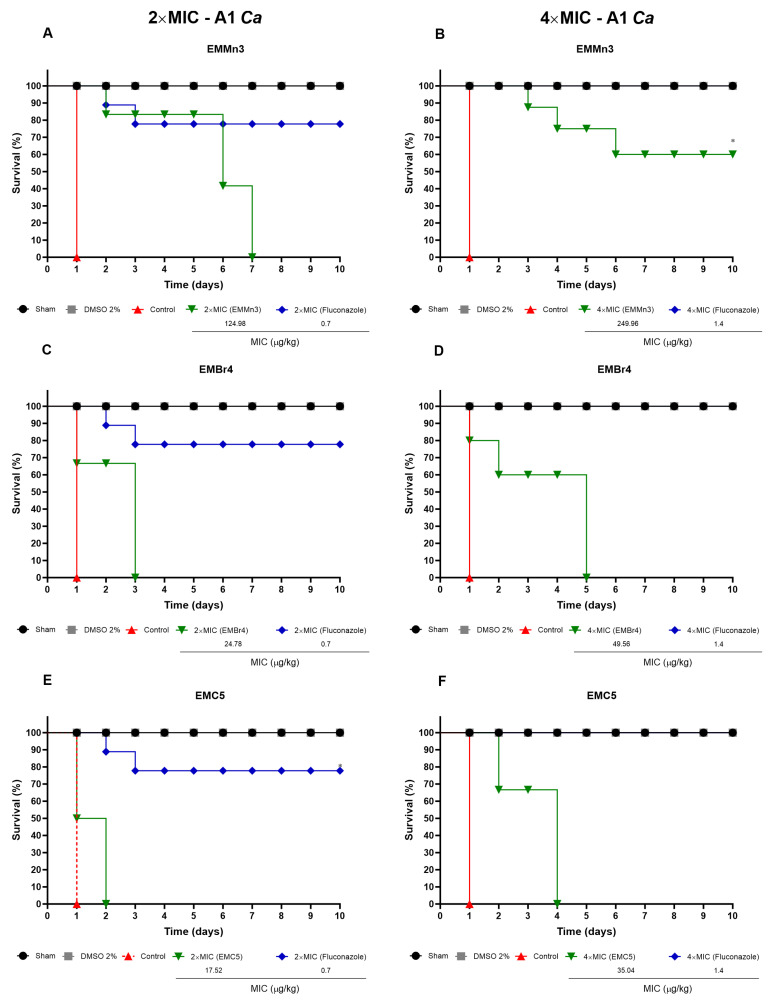
Survival percentage of *Tenebrio molitor* larvae after infection with a clinical isolate of *Candida albicans* (A1) and treatment with *Fusarium* sp. extract fractions EMMn3 (**A**,**B**), EMBr4 (**C**,**D**), and EMC5 (**E**,**F**) at concentrations of 2× MIC (**A**,**C**,**E**) and 4× MIC (**B**,**D**,**F**) (µg/kg). Negative controls received sterile PBS and 2% DMSO. Fluconazole was used as the reference antifungal. Survival was monitored over a 10-day period and analysed using the log-rank test. (*) *p* < 0.05 compared to the control group.

Figure 8 depicts the survival curves of *Tenebrio molitor* larvae infected with a clinical isolate of *Candida albicans* (A1) and treated with the *Fusarium* sp. extract fractions EMMn3, EMBr4, and EMC5 at concentrations of 2× and 4× MIC (µg/kg). Treatment with the EMMn3 fraction (Figure 8A,B) conferred moderate protection, with survival rates of 73.3% at 2× MIC and approximately 60% at 4× MIC. Mortality was concentrated between days 2 and 6 post-treatment.

In contrast, the EMBr4 and EMC5 fractions exhibited superior protective effects. For EMBr4 (Figure 8C,D), survival reached 85% at 2× MIC and 75% at 4× MIC. EMC5 (Figure 8E,F) displayed the most effective profile among all treatment groups, with survival rates of 90% and 87.5% at 2× and 4× MIC, respectively, comparable to the reference antifungal fluconazole.

Negative control groups (infected but untreated or treated with 2% DMSO) exhibited high mortality, particularly within the first 4 days of infection. Conversely, larvae treated with fluconazole consistently maintained survival above 90%, thus validating the reliability and responsiveness of the in vivo model.

## 4. Discussion

Endophytic fungi from plants have emerged as abundant, promising, and valuable sources in the research and development of natural products with pharmacological and/or bioactive potential [43]. Numerous studies have already reported a wide range of biological activities associated with metabolites derived from endophytic fungi [5,44,45,46]. The search for novel antifungal agents among these metabolites represents an innovative approach in the development of this class of drugs, particularly considering the broad availability and low cost of this natural resource—factors that offer significant advantages for its application in antifungal therapy.

Among the annotated compounds, only fusaric acid and cyclosporin A have previously reported antifungal activity in the literature [35,47,48,49,50,51,52,53]. Fusariumin D [33,54] and Cyclo(L-Pro-L-Tyr) [55,56] only exhibit scientific evidence supporting their antibacterial activity. The remaining compounds have not documented antimicrobial and/or antifungal activity.

The antifungal potential of the extract fractions obtained from the endophytic fungus *Fusarium* sp. against *Candida albicans* clinical and reference strains was clearly evidenced in the present study. Fractions EMMn3, EMBr4, and EMC5 exhibited significant fungicidal activity, with MIC values comparable to or slightly higher than fluconazole, yet with MFC/MIC ratios indicative of a fungicidal profile in most cases. These findings are consistent with studies reporting bioactive secondary metabolites produced by endophytic fungi, particularly members of the genus *Fusarium*, which have demonstrated potent inhibitory effects against *Candida* spp. through disruption of membrane integrity and inhibition of ergosterol biosynthesis [54,57,58].

Importantly, the fractions also showed substantial ability to inhibit *C. albicans* biofilm formation and to reduce biomass in mature biofilms. Fraction EMC5, in particular, demonstrated superior activity in both preventive and disruptive biofilm assays. Biofilms are a well-known virulence factor in *Candida* infections, significantly contributing to antifungal resistance and clinical persistence [59,60]. The capacity of these fractions to affect both early and mature biofilms highlights their potential as alternatives or adjuncts to conventional antifungals.

The viability assays with *T. molitor* larvae further corroborated the safety and efficacy of the fractions. At concentrations of up to 2× MIC, none of the fractions induced significant larval mortality, confirming low acute toxicity. This is consistent with other studies using *T. molitor* as an invertebrate model to evaluate the toxicity of plant- or fungus-derived antimicrobials [5,15,61]. Moreover, when infected larvae were treated with the fractions, survival significantly improved, particularly in groups receiving fractions EMBr4 and EMC5. These outcomes suggest that the antifungal activity observed in vitro is reflected in vivo, even in a simplified model of systemic candidiasis.

The survival advantage offered by the fractions was comparable to that observed with fluconazole, especially at higher doses. This similarity is noteworthy considering that biofilm-forming *C. albicans* strains often exhibit decreased susceptibility to azoles. The protective effect seen here may be associated with a multimodal mechanism of action, as described for other Fusarium-derived metabolites [62,63].

The findings of this study reinforce the growing recognition of endophytic fungi as rich reservoirs of pharmacologically relevant secondary metabolites. The ability of *Fusarium* sp. to produce bioactive compounds under modified culture conditions highlights the efficacy of the OSMAC approach when strategically supplemented with halogenated and metal additives. The inclusion of NH_4_Br and MnCl_2_ significantly altered the chemical profile of the fungal extracts, leading to the emergence of unique metabolites not detected under standard conditions, such as (-)-sambutoxin and methoxydianthalexin S. These results support the hypothesis that exogenous chemical stressors can modulate fungal biosynthetic pathways and induce cryptic metabolite expression.

Collectively, these findings indicate that the tested fractions, particularly EMBr4 and EMC5, possess robust antifungal activity against *C. albicans,* including in biofilm and in vivo infection models. Their low toxicity, coupled with broad-spectrum efficacy, reinforces the potential of endophytic fungi as valuable sources for novel antifungal agents. This study strengthens the argument for exploring microbial endophytes in the discovery of next-generation antifungal agents, particularly for challenging infections involving biofilm-forming pathogens. Further chemical characterisation and mechanistic studies are warranted to elucidate the specific compounds responsible for the observed effects and their possible synergism with existing antifungal drugs.

## 5. Conclusions

This study confirms the antifungal potential of extract fractions from the endophytic fungus *Fusarium* sp. associated with *Dizygostemon riparius*. The fractions EMBr4 and EMC5 exhibited fungicidal activity against *Candida albicans*, including biofilm-forming strains, with efficacy comparable to fluconazole. These fractions also significantly inhibited both early-stage and mature biofilms. In vivo assays using *Tenebrio molitor* larvae demonstrated low acute toxicity and improved survival in infected groups, reinforcing the therapeutic potential. Chemical analyses identified known antifungal metabolites such as fusaric acid and cyclosporin A, alongside other compounds with no previously reported antimicrobial activity. These findings highlight endophytic fungi as promising sources of novel antifungal agents and support further investigation into their active constituents and mechanisms of action.

## Figures and Tables

**Table 1:** Annotated compounds detected by UPLC-ESI-QTOF-MS in positive and negative modes from fractions EMC5, EMBr4, and EMMn3 derived from *Fusarium* species.

Nª	RT (min)	Observed Mass (Da)	Calculated Mass (Da)	∆ (ppm)	Annotated	EMC5	EMBr4	EMMn3	Ref
						(Control)	(NH_4_Br)	(MnCl_2_)	
1	2.4	220.1182 ^a^	220.1193	−4.99	Zeatin (cytokinins)	X	--	--	[28]
2	3.32	180.1026 ^a^	180.1019	3.88	Fusaric acid	X	--	--	[29]
3	3.83	211.1441 ^a^	211.1441	0.00	L-Leucyl-L-proline lactam	X	--	--	[30]
4	8.93	265.1805 ^a^	265.1798	2.64	4-methoxy-6-((E)-4,6- dimethyloct-2-en-2-yl)- 2H-pyran-2-one	X	--	--	[31]
5	11.09	1202.8492 ^a^	1202.8486	−0.58	Cyclosporin A	X	--	--	[32]
6	8.94	265.1805 ^a^	265.1798	2.63	Fusariumin D	X	--	--	[33]
7	10.97	397.3470 ^a^	397.3465	1.25	Ergosterol	X	--	--	[34]
8	4.29	261.1234 ^a^	261.1234	0.00	Cyclo(L-Pro-L-Tyr)	--	X	--	[35]
9	8.78	454.2937 ^a^	454.2952	−3.30	(−)-Sambutoxin	--	X	--	[36]
10	6.55	221.1168 ^b^	221.1183	−2.26	Dehydrovomifoliol	--	X	--	[37]
11	7.3	293.1781 ^b^	293.1758	7.84	5-hydroxy-1-(4-hydroxy-3-methoxyphenyl)decan-3-one	--	--	X	[38]
12	7.3	286.0704 ^a^	286.0710	−2.09	Bostrycoidin	--	--	X	[39]
13	7.72	270.0767 ^a^	270.0761	2.22	Methoxydianthalexin S	--	--	X	[40]
14	10.29	425.2144 ^a^	425.2170	−6.11	HT-2 Toxin	--	--	X	[41]
15a	6.53	295.2267 ^a^	295.2268	−0.33	(1S,2R)-3-Oxo-2-(2Z-pentenyl) cyclopentane-1-octanoic acid	--	--	X	[42]
15b	6.53	295.2267 ^a^	295.2268	−0.33	(1S,2S)-3-Oxo-2-(2Z-pentenyl) cyclopentane-1-octanoic acid	--	--	X	[42]

Legend: RT = retention time (min); EMC5 = control methanolic fraction; EMBr4 = methanolic fraction with ammonium bromide; EMMn3 = methanolic fraction with manganese; ^a^ = positive mode; ^b^ = negative mode, X = present, -- = not present.

**Table 2:** Minimum inhibitory (MIC) and fungicidal (MFC) concentrations of extract fractions from the endophytic fungus *Fusarium* sp. against *Candida albicans* strains.

	Extract Fractions from the Endophytic Fungus *Fusarium* sp.									Antifungal
	EMMn3			EMBr4			EMC5			Fluconazole
*Candida albicans* Strain	MIC ^a^	MFC ^a^	MFC/MIC Ratio	MIC	MFC	MFC/MIC Ratio	MIC	MFC	MFC/MIC Ratio	MIC
*Ca* (ATCC 90028) ^B^	11.04	44.19	4.00	4.19	15.62	3.17	15.61	55.67	3.56	0.56
*Ca* (ATCC 10231) ^B^	15.62	44.19	2.82	99.21	353.55	3.56	111.36	445.44	4	1.41
A1 ^C^ *Ca*	62.49	157.49	2.52	12.39	27.83	4.00	8.76	24.80	2.83	0.35
A2 *Ca*	31.25	124.91	3.99	31.24	78.74	2.52	35.89	62.5	1.74	0.89
A3 *Ca*	280.61	561.23	0.49	35.07	140.30	4.00	55.67	125	2.24	1

(^a^) Values are expressed as µg/mL. (^B^) ATCC^®^ (American Type Culture Collection). (^C^) A1–A3: clinical strain.

## Data Availability

The original contributions presented in the study are included in the article and supplementary material. Further inquiries can be directed to the corresponding author.

## Supplementary Materials

The following supporting information can be downloaded at https://www.mdpi.com/article/10.3390/metabo15070451/s1. Figure S1: Total Ion Chromatogram (TIC) in positive mode of the EMC5 fraction; Figure S2: Total Ion Chromatogram (TIC) in negative mode of the EMC5 fraction; Figure S3: Total Ion Chromatogram (TIC) in positive mode of the EMMN3 fraction; Figure S4: Total Ion Chromatogram (TIC) in negative mode of the EMMN3 fraction; Figure S5: Total Ion Chromatogram (TIC) in positive mode of the EMBr4 fraction; Figure S6: Total Ion Chromatogram (TIC) in negative mode of the EMBr4 fraction; Figure S7: Base ion chromatogram in positive mode with *m*/*z* = 220.1182; Figure S8: Base ion chromatogram in positive mode with *m*/*z* = 180.1026; Figure S9: Base ion chromatogram in positive mode with *m*/*z* = 211.1441; Figure S10: Base ion chromatogram in positive mode with *m*/*z* = 265.1805; Figure S11: Base ion chromatogram in positive mode with *m*/*z* = 1202.8492; Figure S12: Base ion chromatogram in positive mode with *m*/*z* = 265.1805; Figure S13: Base ion chromatogram in positive mode with *m*/*z* = 397.3470; Figure S14: Base ion chromatogram in positive mode with *m*/*z* = 261.1234; Figure S15: Base ion chromatogram in positive mode with *m*/*z* = 454.2937; Figure S16: Base ion chromatogram in negative mode with *m*/*z* = 221.1168; Figure S17: Base ion chromatogram in negative mode with *m*/*z* = 293.1781; Figure S18: Base ion chromatogram in positive mode with *m*/*z* = 286.0704; Figure S19: Base ion chromatogram in positive mode with *m*/*z* = 270.0767; Figure S20: Base ion chromatogram in positive mode with *m*/*z* = 425.2144; Figure S21: Base ion chromatogram in positive mode with *m*/*z* = 295.2267; Table S1: Fractions and respective solvent systems obtained from the methanolic extract (E.MeOH) of the control (EMC), NH_4_Br (EMBr) and MnCl_2_ (EMMn) media of *Fusarium* sp.
