# Supplementary material for: Assessment of Metabolic Alterations Induced by Halogenated Additives and Antifungal Activity of Extracts from the Endophytic Fungus Fusarium sp. Associated with Dizygostemon riparius (Plantaginaceae)
Source: Metabolites. 2025 Jul 4;15(7):451. doi: 10.3390/metabo15070451 (PMC12300354; doi:10.3390/metabo15070451)

Table S1: Fractions and respective solvent systems obtained from the methanolic extract (E.MeOH) of the control (EMC), NH_4Br (EMBr) and $MnCl_2$ (EMMn) media of *Fusarium* sp.

Eluents	Fractions		
	Sample code (EMC)	Sample code (EMBr)	Sample code (EMMn)
**Hex/EtOAc (1:1)**	EMC1	EMBr1	EMMn1
**EtOAc 100%**	EMC2	EMBr2	EMMn2
**EtOAc/MeOH (9:1)**	EMC3	EMBr3	**EMMn3***
**EtOAc/MeOH (7:3)**	EMC4	**EMBr4***	EMMn4
**EtOAc/MeOH (1:1)**	**EMC5***	EMBr5	EMMn5
**EtOAc/MeOH (7:3)**	EMC6	EMBr6	EMMn6
**EtOAc/MeOH (8:2)**	EMC7	EMBr7	EMMn7
**MeOH 100%**	EMC8	EMBr8	EMMn8

Legend: Hex (hexane), EtOAc (ethyl acetate), MeOH (methanol), *(fractions studied)

Figure S1: Total Ion Chromatogram (TIC) in positive mode of the EMC5 fraction.

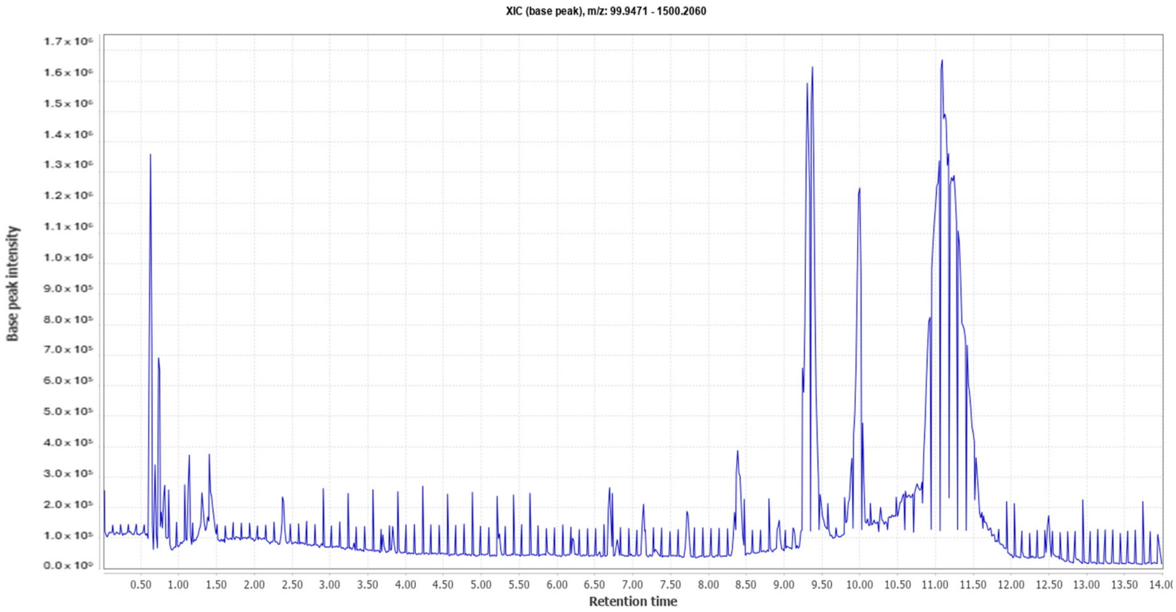

Figure S2: Total Ion Chromatogram (TIC) in negative mode of the EMC5 fraction.

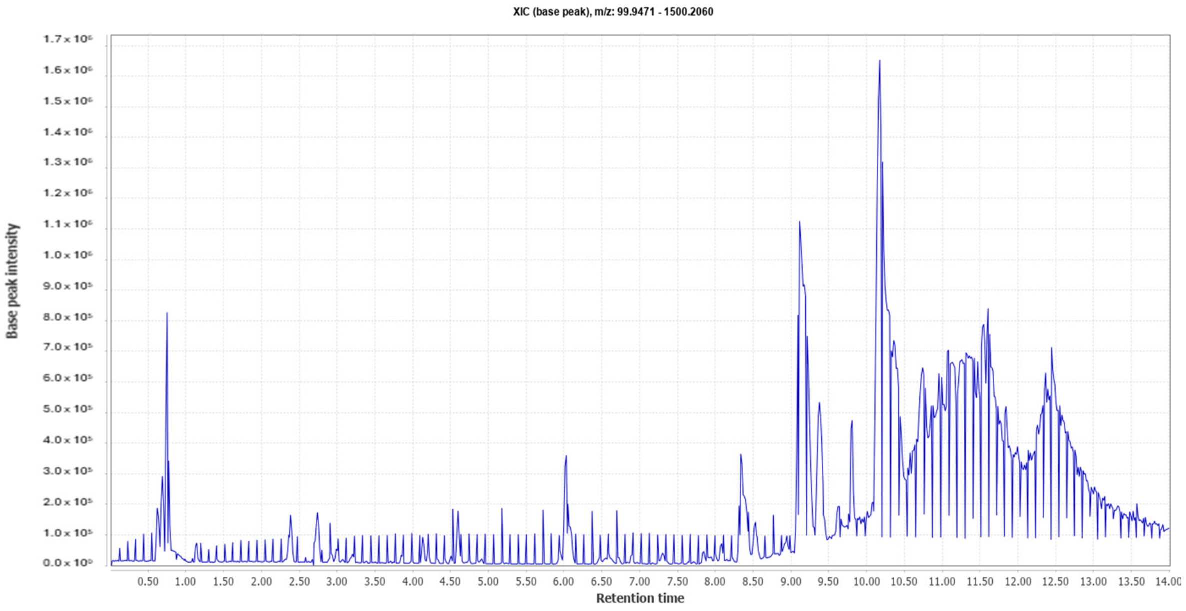

Figure S3: Total Ion Chromatogram (TIC) in positive mode of the EMMN3 fraction.

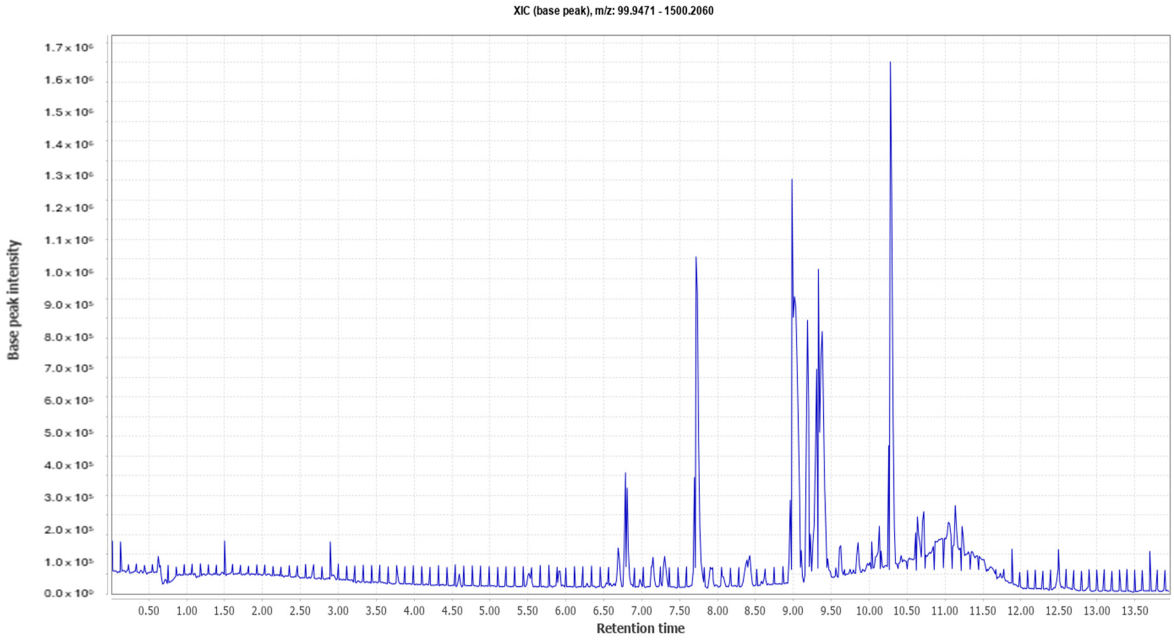

Figure S4: Total Ion Chromatogram (TIC) in negative mode of the EMMN3 fraction.

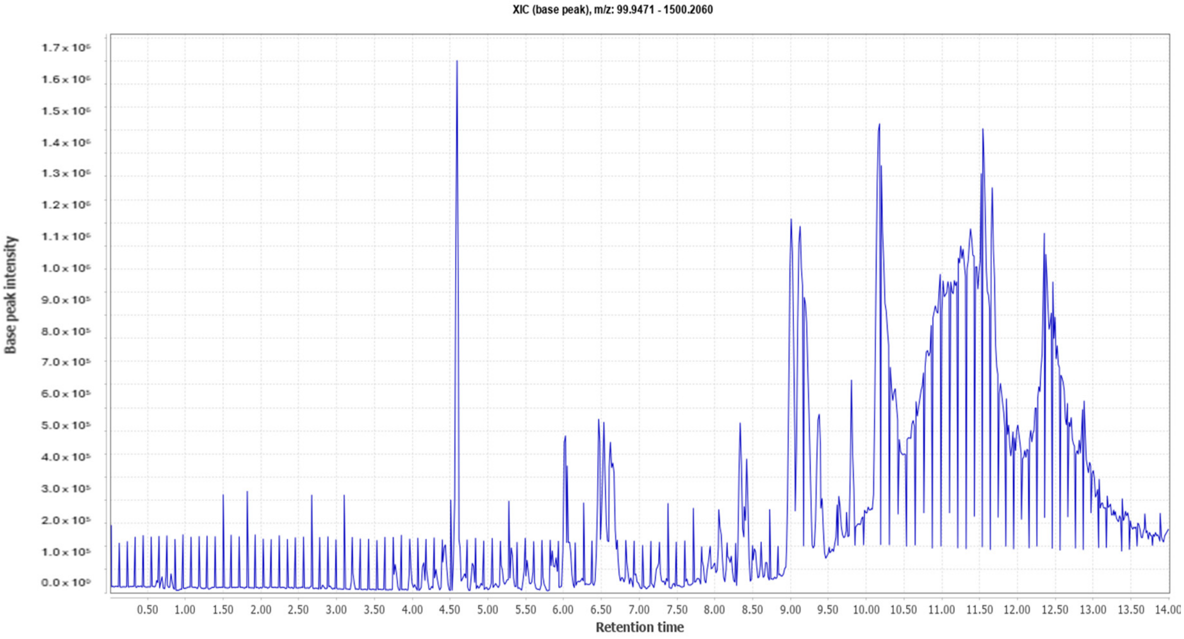

Figure S5: Total Ion Chromatogram (TIC) in positive mode of the EMBr4 fraction.

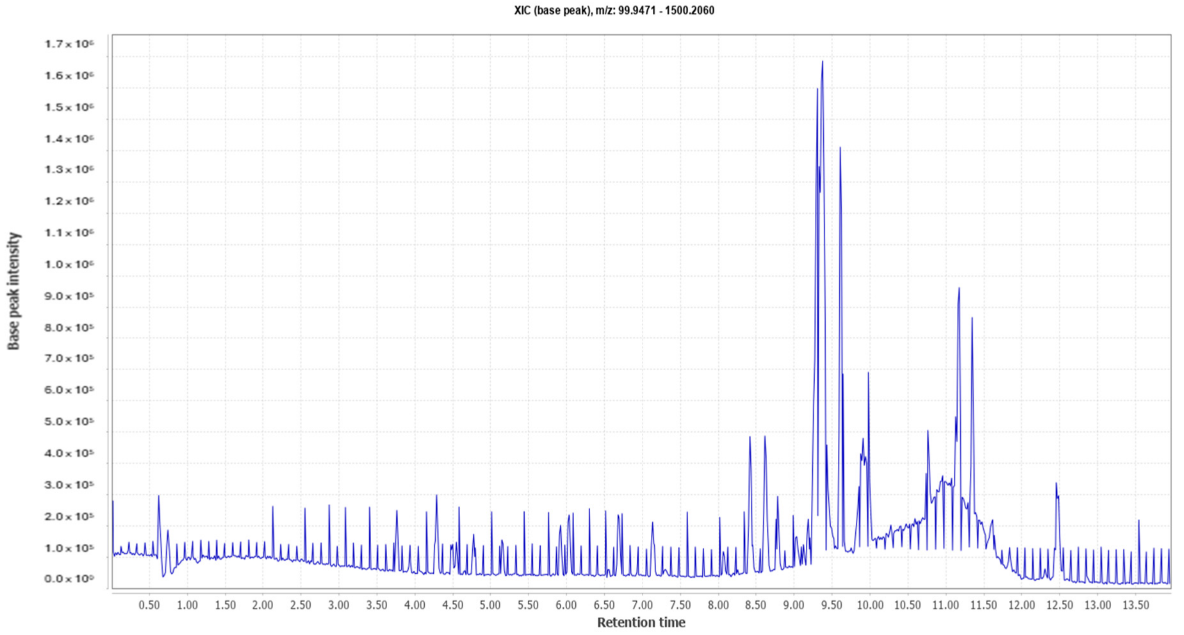

Figure S6: Total Ion Chromatogram (TIC) in negative mode of the EMBr4 fraction.

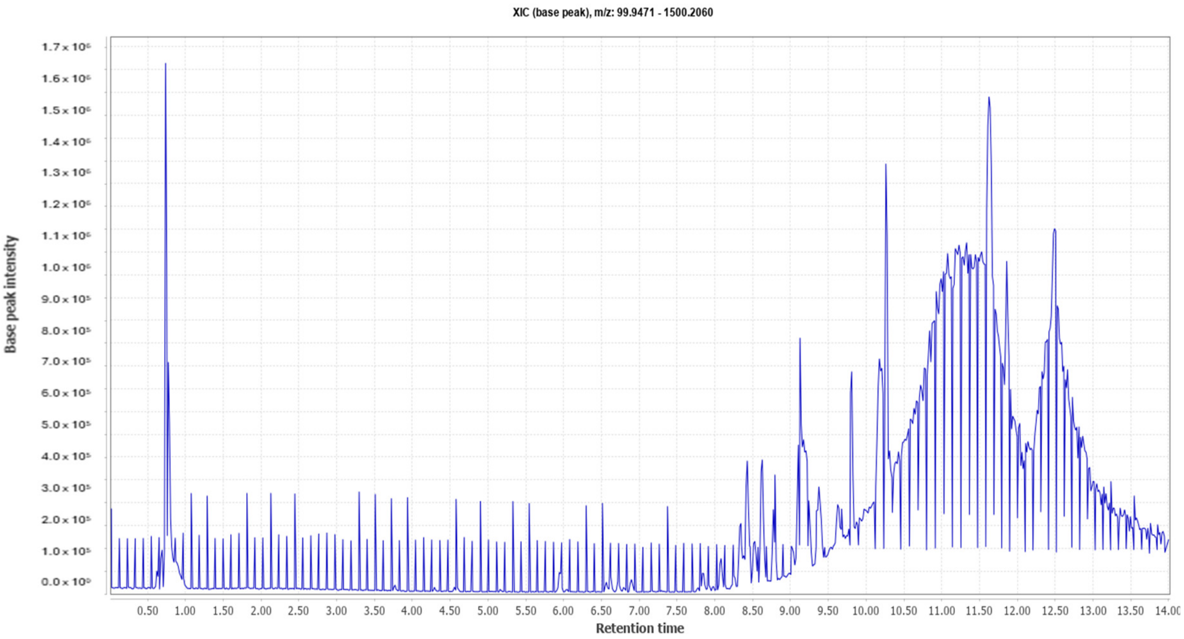

Figure S7: Base ion chromatogram in positive mode with m/z = 220.1182.

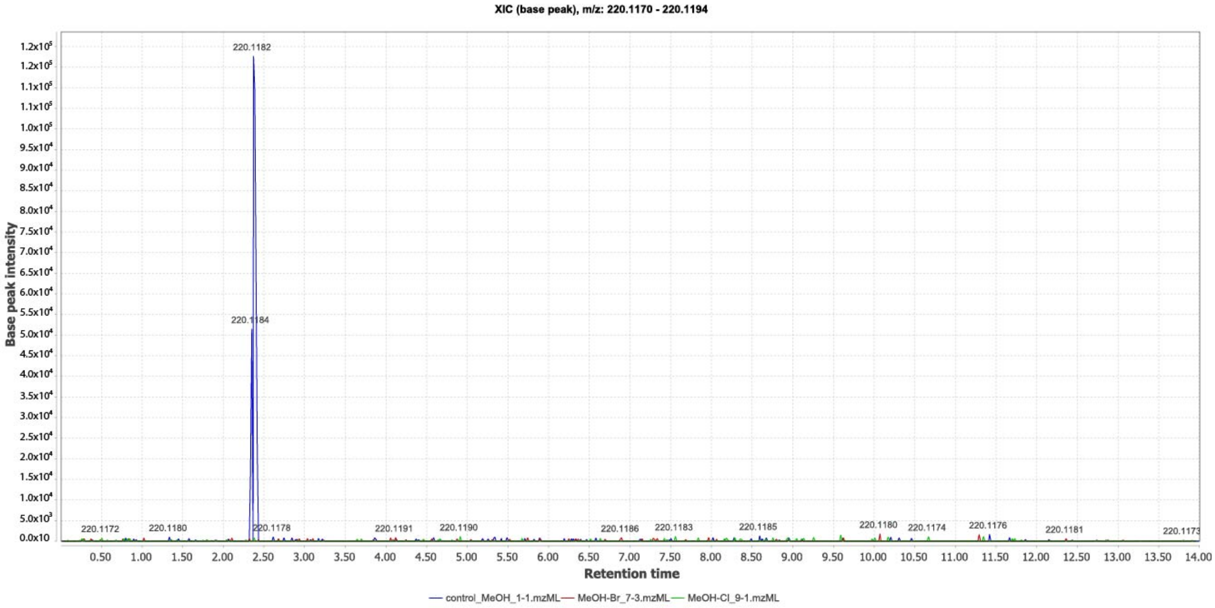

Figure S8: Base ion chromatogram in positive mode with m/z = 180.1026.

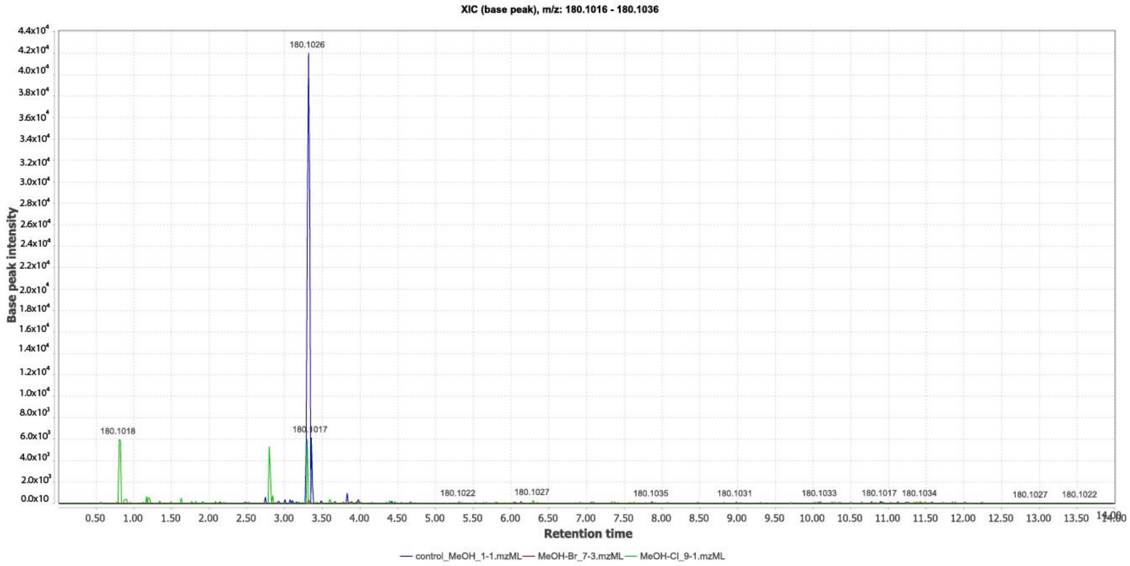

Figure S9: Base ion chromatogram in positive mode with m/z = 211.1441.

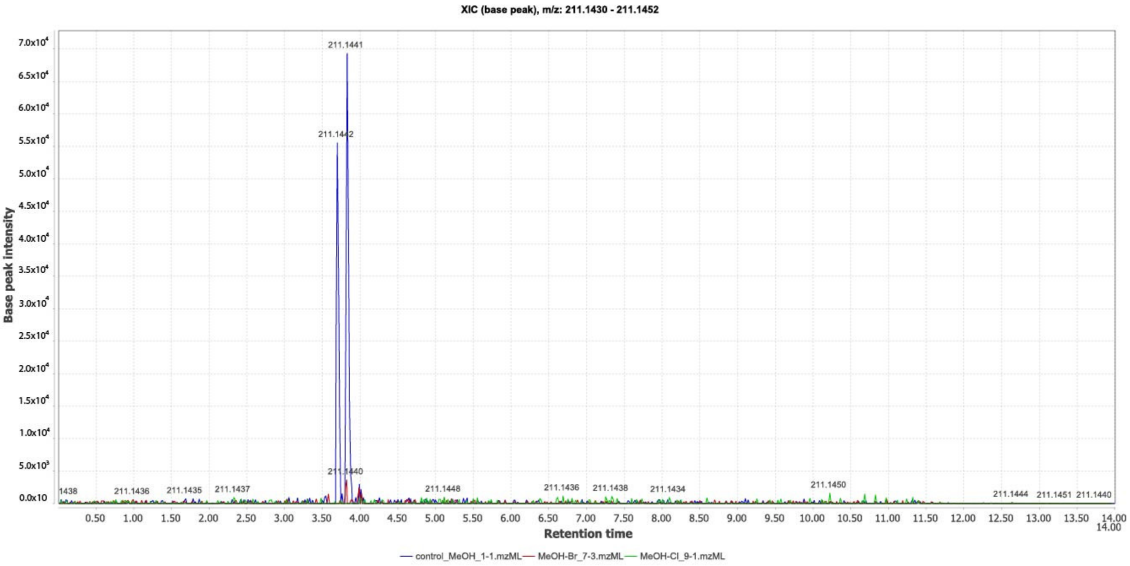

Figure S10: Base ion chromatogram in positive mode with m/z = 265.1805.

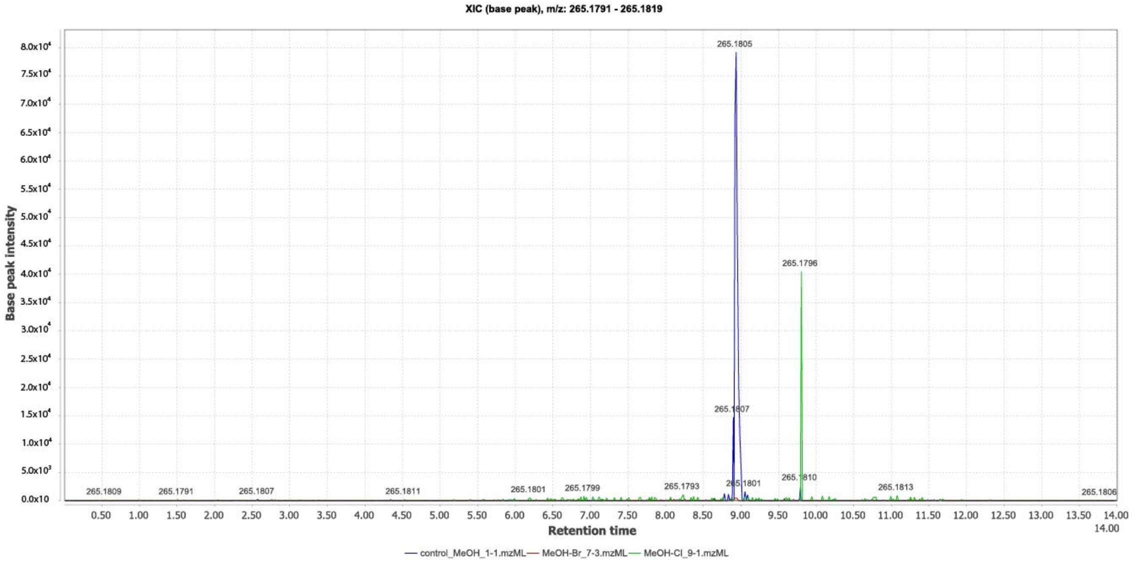

Figure S11: Base ion chromatogram in positive mode with m/z = 1202.8492.

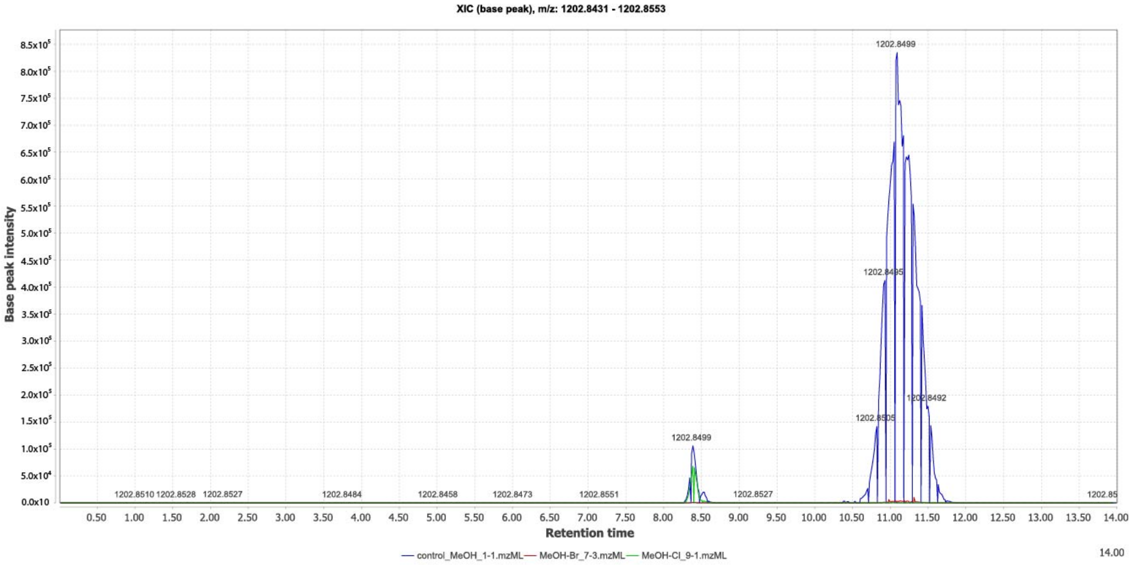

Figure S12: Base ion chromatogram in positive mode with m/z = 265.1805.

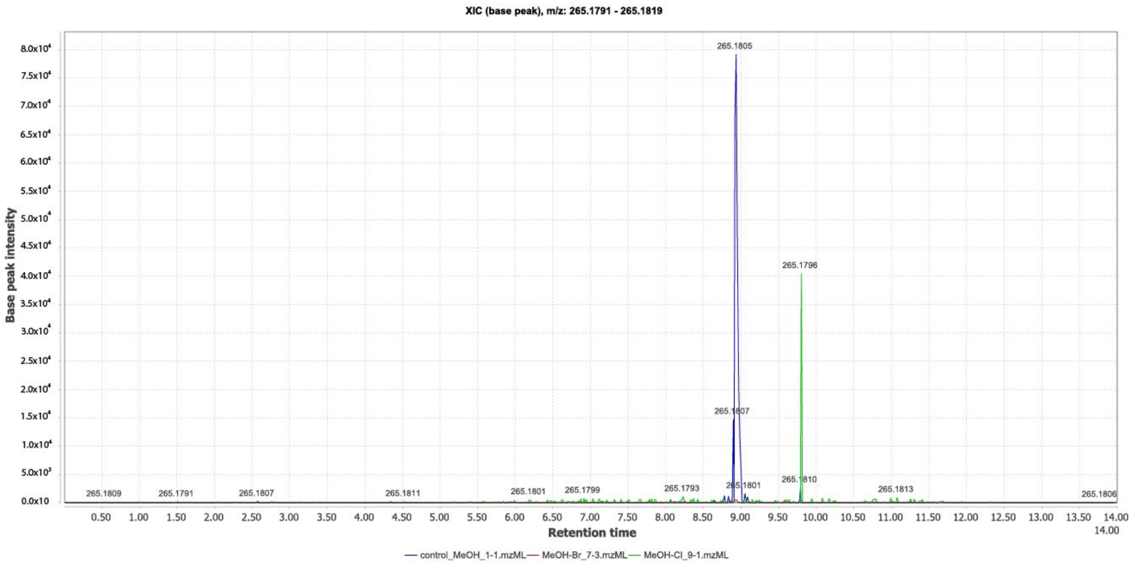

Figure S13: Base ion chromatogram in positive mode with m/z = 397.3470.

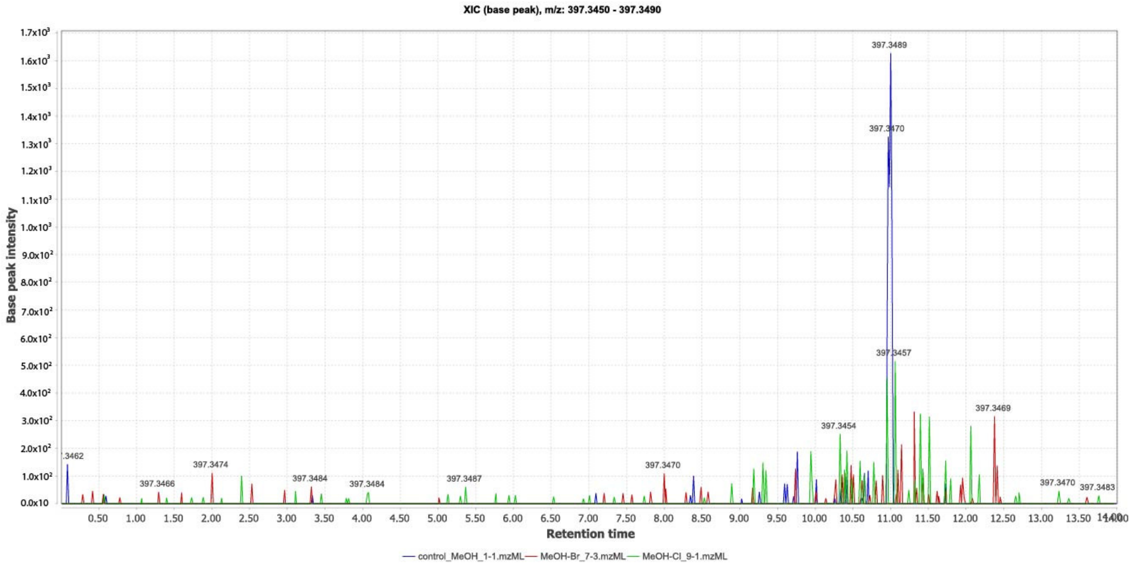

Figure S14: Base ion chromatogram in positive mode with m/z = 261.1234.

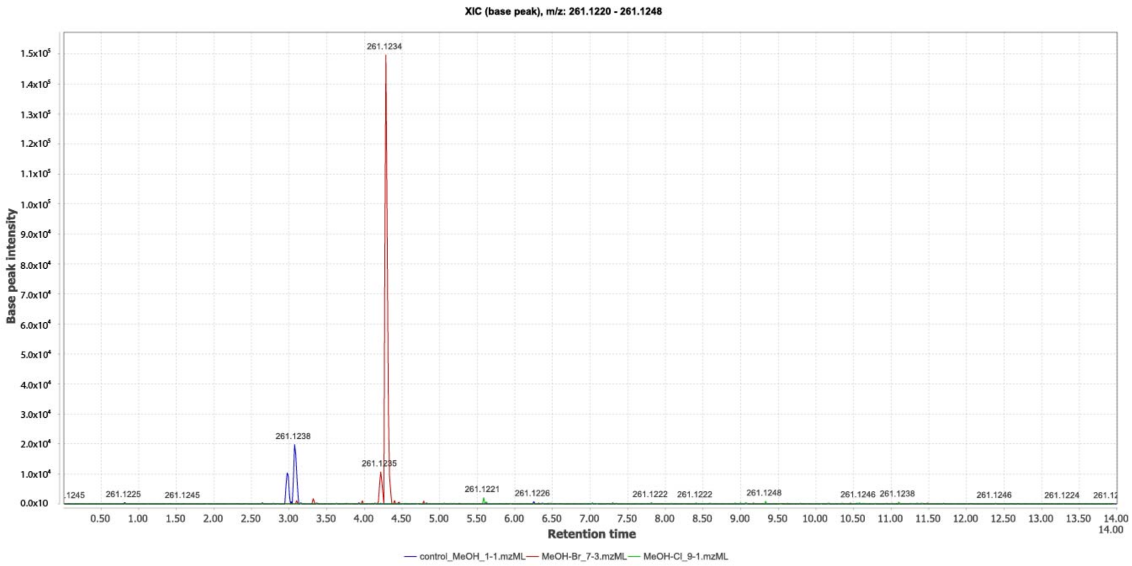

Figure S15: Base ion chromatogram in positive mode with m/z = 454.2937.

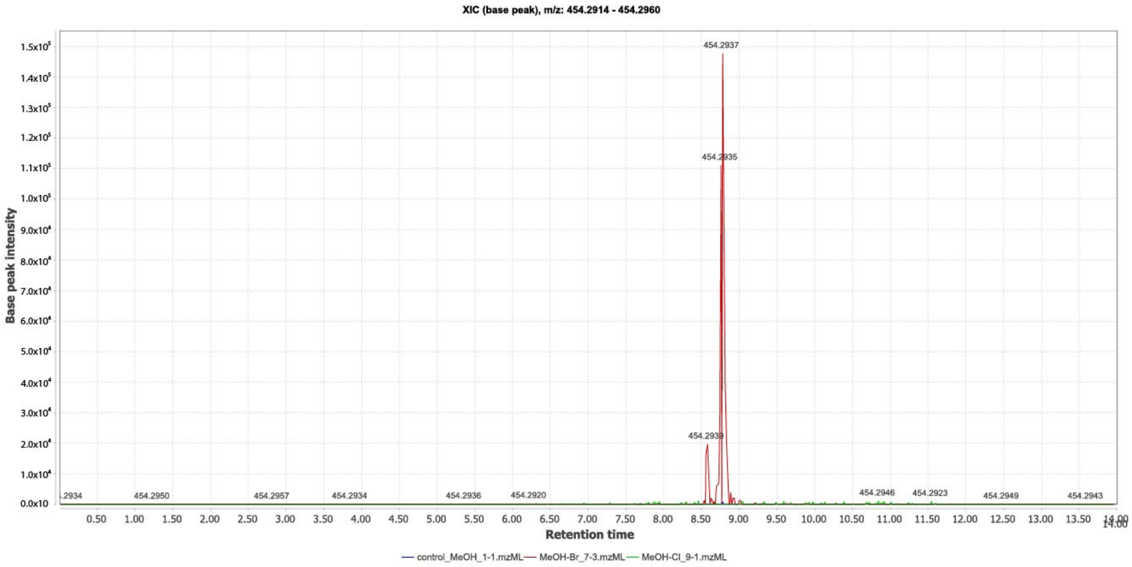

Figure S16: Base ion chromatogram in negative mode with m/z = 221.1168.

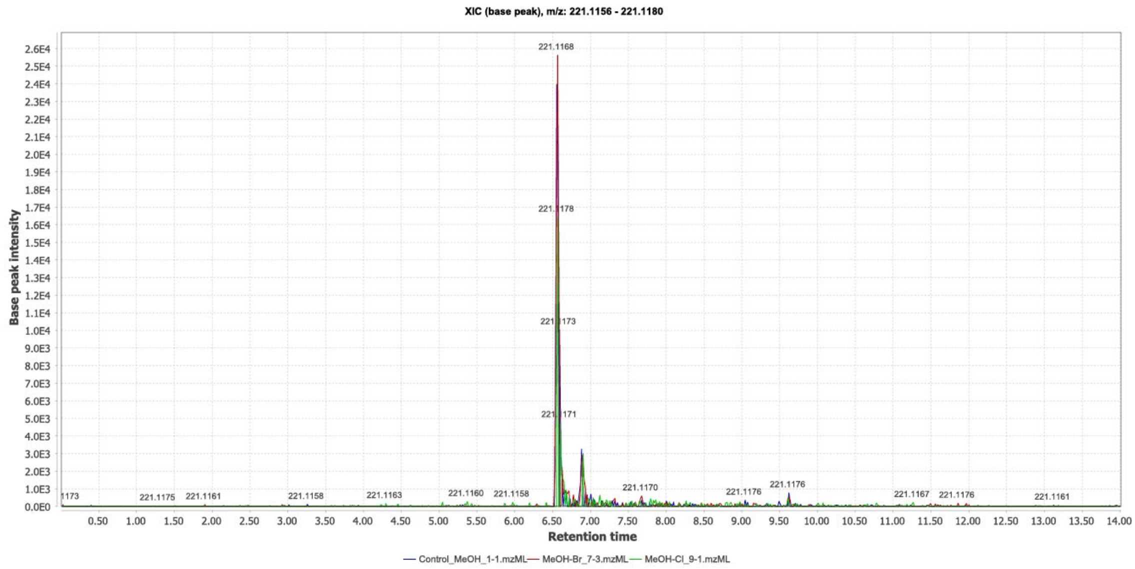

Figure S17: Base ion chromatogram in negative mode with m/z = 293.1781.

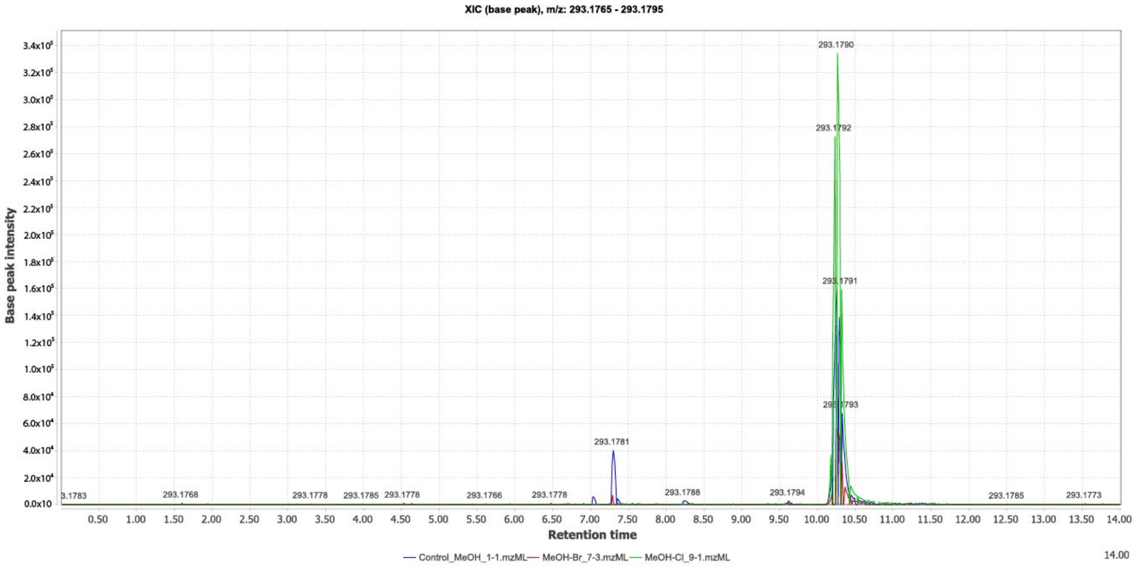

Figure S18: Base ion chromatogram in positive mode with m/z = 286.0704.

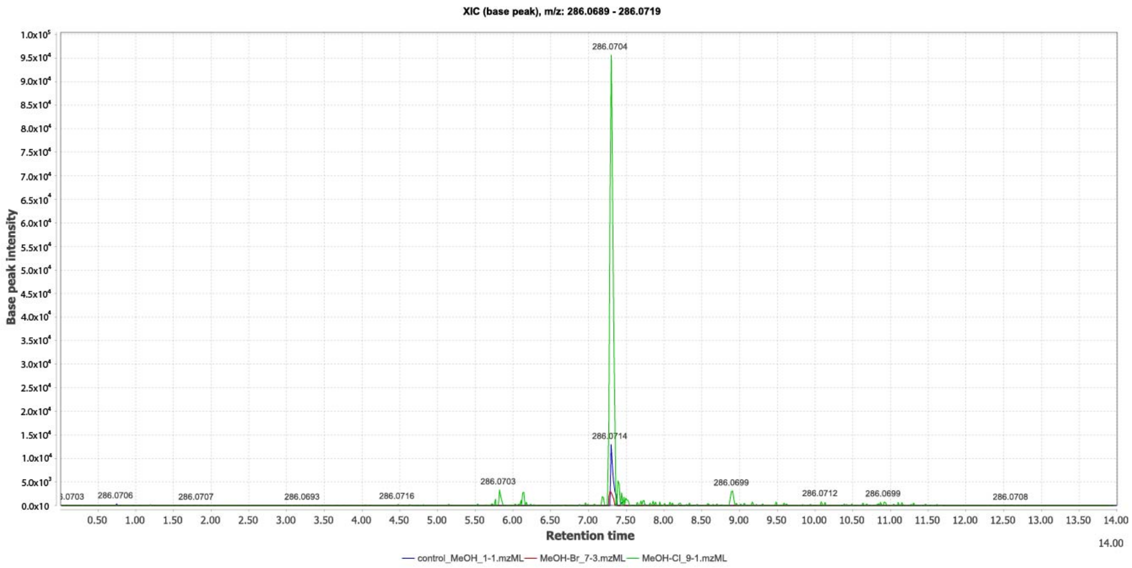

Figure S19: Base ion chromatogram in positive mode with m/z = 270.0767.

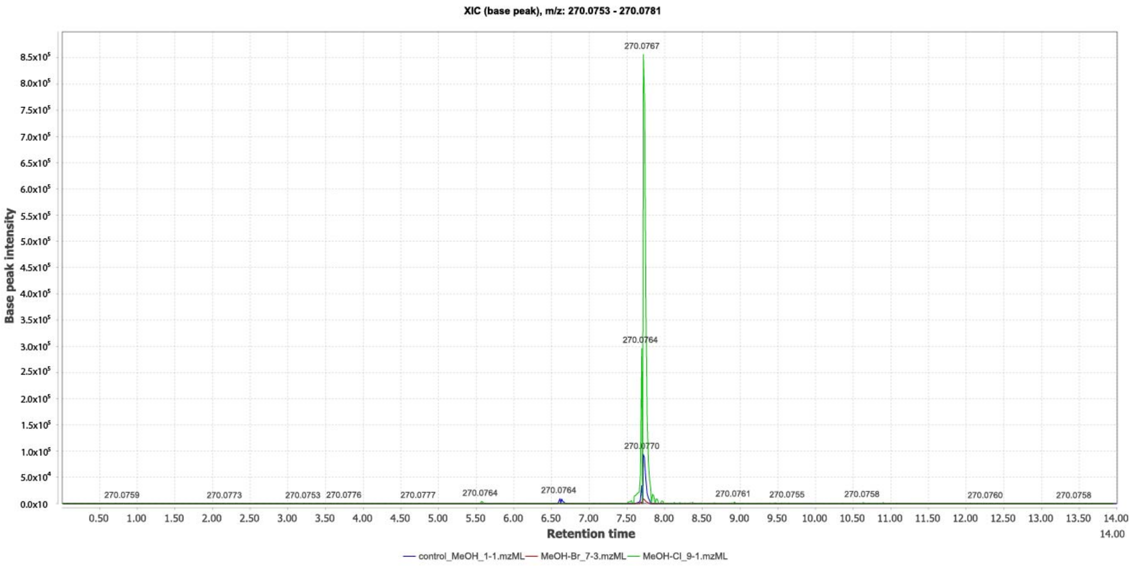

Figure S20: Base ion chromatogram in positive mode with m/z = 425.2144.

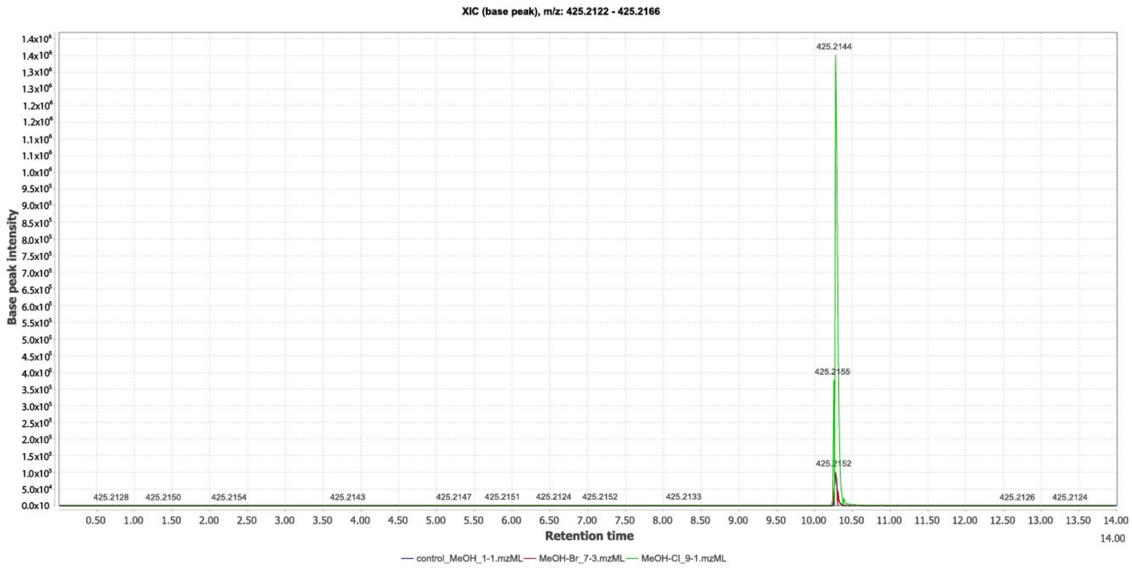

Figure S21: Base ion chromatogram in positive mode with m/z = 295.2267.

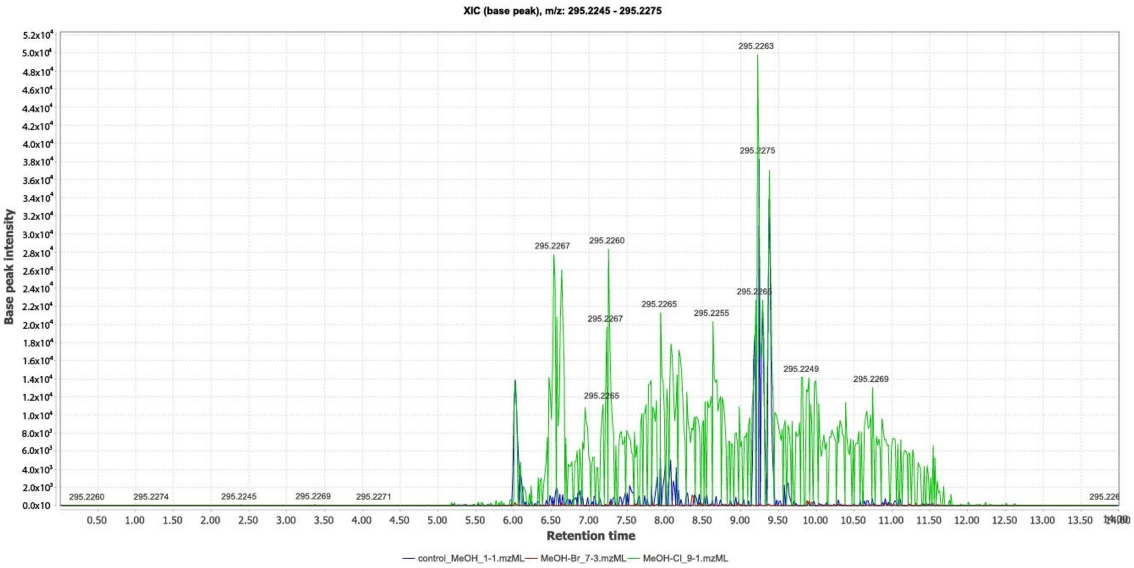

Supplement: Supplementary file 1 [file metabolites-15-00451-s001.zip › metabolites-3721081-supplementary.pdf]
